# Regulatory Effects of *Betonica macrantha* Extract, Known as Mountain Tea, on miRNA Expression and Its Pharmacokinetic Properties

**DOI:** 10.1002/fsn3.70341

**Published:** 2025-05-25

**Authors:** Muhammed Emin Sarı, Emine Incilay Torunoğlu, Zeynep Betül Sarı, Erdi Can Aytar, Abidin Gümrükçüoğlu, Alper Durmaz, Gamze Demirel

**Affiliations:** ^1^ Department of Medical Biology, Faculty of Medicine Necmettin Erbakan University Konya Türkiye; ^2^ Department of Medical Biochemistry, Faculty of Medicine Necmettin Erbakan University Konya Türkiye; ^3^ Department of Basic Medical Sciences, Medical Biology, Faculty of Medicine Ankara Yıldırım Beyazıt University Ankara Türkiye; ^4^ Department of Horticulture, Faculty of Agriculture Usak University Uşak Türkiye; ^5^ Medicinal‐Aromatic Plants Application and Research Center Artvin Çoruh University Artvin Türkiye; ^6^ Ali Nihat Gökyiğit Botanical Garden Application and Research Center Artvin Çoruh University Artvin Türkiye; ^7^ Department of Nutrition and Dietetics, Akşehir Kadir Yallagöz School of Health Selçuk University Konya Türkiye

**Keywords:** *B. macrantha*, GC–MS analysis, HPLC‐DAD analysis, MDA‐MB‐231 cancer cell line, miRNA expression, molecular docking

## Abstract

The aim of this study is to investigate the anticancer potential of *Betonica macrantha* extract on MDA‐MB‐231 breast cancer cells, its regulatory effects on miRNA expression, the content analysis of its phytochemical components, and the roles of these compounds in the regulation of miRNA expression through pathways, as well as to examine its pharmacokinetic profiles. The *B. macrantha* plant was extracted with methanol. The obtained extracts were analyzed for phytochemical components using gas chromatography–mass spectrometry (GC–MS) and high‐performance liquid chromatography (HPLC‐DAD) techniques. Biological activity was assessed using the MTT assay on the MDA‐MB‐231 breast cancer cell line, while miRNA expression was measured by RT‐PCR. Pharmacokinetic properties were calculated using ADMETSAR3 software, and molecular interactions were investigated through AutoDock Vina simulations. GC–MS analysis of *B. macrantha* extract identified 42 volatile compounds, with 1S‐α‐pinene, humulene, and caryophyllene being the most abundant. HPLC‐DAD analysis detected 18 bioactive compounds, including catechin, oleuropein, and rutin. The extract inhibited the viability of MDA‐MB‐231 breast cancer cells in a dose‐dependent manner, with an IC_50_ value of 0.8 mg/mL. Furthermore, upregulation of miR‐19, miR‐20a, miR‐126, and miR‐200c miRNAs was observed in MDA‐MB‐231 cells, while these miRNAs were downregulated in healthy cells. ADMET analysis revealed that α‐pinene, caryophyllene, and catechin exhibited high bioavailability, absorption, and distribution properties, while oleuropein and rutin showed limited absorption and bioavailability. Molecular docking studies demonstrated the potential binding interactions of these compounds with key target proteins involved in cancer progression. Consequently, *B. macrantha* presents significant potential as a valuable natural source for cancer therapy through its anticancer activity, modulation of miRNA expression, and interaction with cancer‐associated proteins.

## Graphical abstract

*Betonica macrantha* methanol extract exhibited anticancer activity against MDA‐MB‐231 breast cancer cells (IC_50_: 0.8 mg/mL), upregulating miR‐19, miR‐20a, miR‐126, and miR‐200c. GC–MS and HPLC analyses identified key bioactive compounds, including α‐pinene, caryophyllene, and catechin, which showed favorable pharmacokinetics and target interactions. These findings suggest *B. macrantha* as a promising candidate for natural cancer therapeutics via miRNA modulation and protein binding.

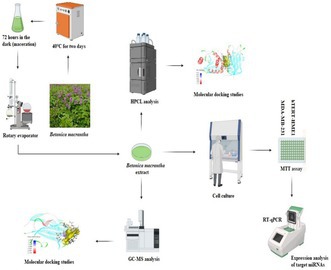

## 1 Introduction

Today, breast cancer accounts for approximately 25% of all cancer diagnoses among women, with over 2.3 million new cases reported annually, reflecting both high mortality and prevalence (Fidler‐Benaoudia et al. 2020; Harbeck et al. 2019). The effectiveness of current chemotherapy and radiotherapy protocols is significantly hindered by multidrug resistance mechanisms and severe side effects, highlighting the urgent need for the development of more selective and safer targeted therapeutic strategies. In this context, phytochemical compounds of plant origin have attracted growing scientific interest in recent years due to their anti‐proliferative effects, ability to induce apoptosis, and potential to modulate drug resistance pathways (Mladenova et al. 2022). Despite this interest, comprehensive data on the anticancer potential of plant species endemic to the Turkish flora such as *Betonica macrantha* remain limited. There is a significant knowledge gap regarding the influence of such plant‐derived compounds on microRNA (miRNA) regulation, which plays a pivotal role in the molecular mechanisms underlying cancer development, progression, and therapy resistance. Bridging this gap could pave the way for novel plant‐based therapeutic approaches in precision oncology.

**FIGURE 1:**
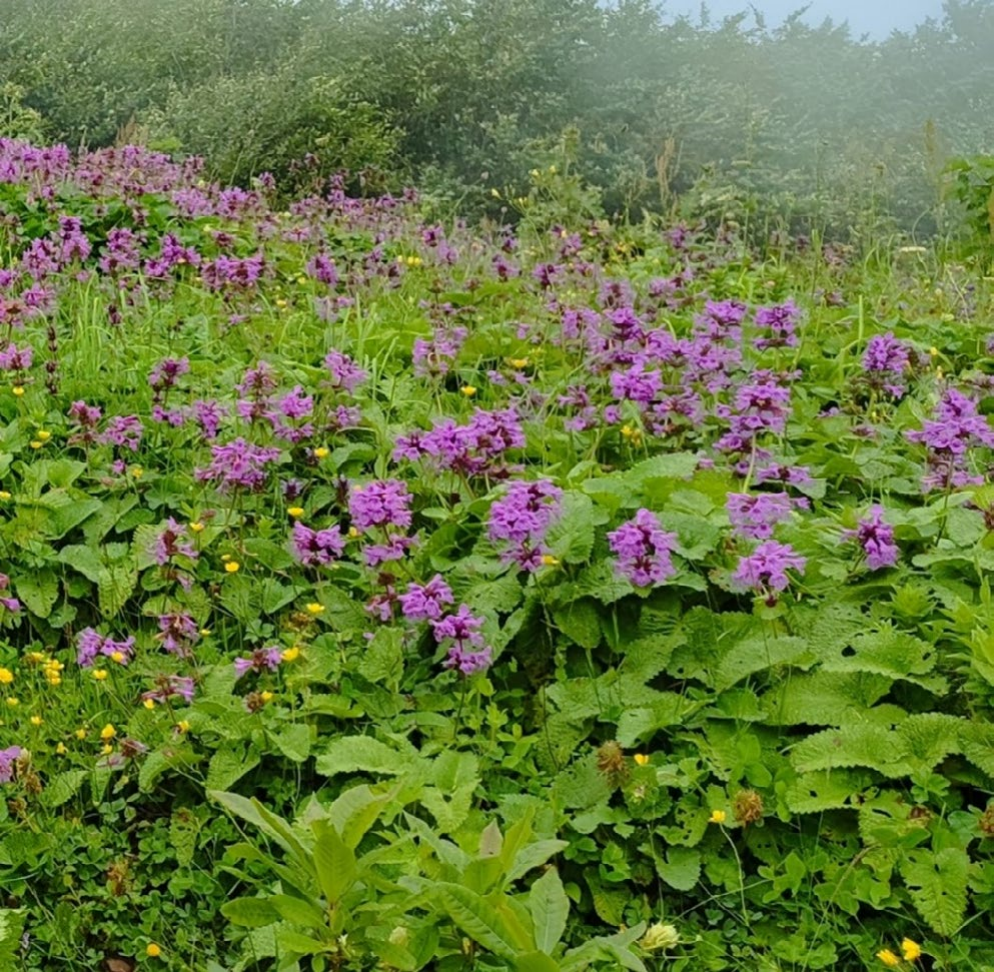
*Betonica macrantha*.

*B. macrantha* K. Koch [syn. *Stachys macrantha* “mountain tea” (K. Koch)] is a perennial herbaceous species within the Lamiaceae family, first described in Linnaea in 1849 (Figure 1). This taxon, which belongs to the genus *Betonica* comprising approximately 372 species globally (POWO 2024), is native to moist, high‐altitude regions of the Caucasus, northeastern Türkiye, and northwestern Iran. Phytochemical analyses have revealed that *B. macrantha* is particularly rich in flavonoids, iridoids, and phenolic acids (Davis 1982; Çalış et al. 1992). *Stachys* species are widely used in traditional medicine to treat digestive disorders, respiratory diseases, and inflammatory conditions. *B. macrantha* is known as “mountain tea” (Özcan and Acet 2022) and its infusions are traditionally used for the treatment of skin and gastrointestinal disorders (Tomou et al. 2020). Antitumor and immunomodulatory findings reported in other *Betonica* species have highlighted the biomedical potential of this genus (Tomou et al. 2020; Mladenova et al. 2022). Additionally, it has been shown that plant‐derived compounds can modulate microRNA (miRNA) expression, targeting both tumor suppressor and oncomiR pathways offering innovative opportunities for both diagnosis and therapy (Sumaira et al. 2024). However, the regulatory effects of *B. macrantha* extracts on oncomiRs (miR‐19b, miR‐20a, miR‐155) and tumor suppressor miRNAs (miR‐126, miR‐200c) in triple‐negative MDA‐MB‐231 breast cancer cells and hTERT‐immortalized human epithelial cells have not yet been investigated.

Recent developments in molecular biology have brought attention to the critical functions that microRNAs (miRNAs) play in the development of cancer. miRNAs are tiny, non‐coding RNA molecules that post‐transcribe to control gene expression. They are around 22 nucleotides long. They do this by attaching themselves to the 3′ untranslated regions (UTRs) of target messenger RNAs (mRNAs), which either cause translational inhibition or mRNA destruction (Condrat et al. 2020; Sabit et al. 2021). miRNAs are crucial for preserving cellular homeostasis and halting the development of tumors because they affect important cellular functions like proliferation, apoptosis, angiogenesis, and metastasis.

The onset, development, and resistance to treatment of breast cancer have all been linked to aberrant miRNA expression. It is well‐recognized that tumor‐suppressive miRNAs, including the let‐7 family, miR‐126, and miR‐200c, prevent cancerous processes and preserve cellular equilibrium. These defenses are compromised by their downregulation in breast cancer, which increases tumor aggressiveness and spread. On the other hand, oncogenic miRNAs, also known as oncomiRs, such as miR‐19, miR‐20a, and miR‐155, increase pro‐tumorigenic pathways and target tumor suppressor genes to accelerate the growth of tumors (Tristán‐Ramos et al. 2020). Because of their dual function, miRNAs are useful as therapeutic targets as well as diagnostic indicators.

Experimental models, such as telomerase‐immortalized human epithelial cells (hTERT) and the metastatic breast cancer cell line MDA‐MB‐231, have been created to better understand the functional functions of miRNAs in breast cancer. Derived from a metastatic TNBC tumor, MDA‐MB‐231 cells are utilized extensively to investigate advanced cancer processes, such as metastasis and resistance to treatment, because of their extremely aggressive activity (Hu et al. 2022). hTERT cells, on the other hand, are a non‐cancerous model that closely resembles the activity of normal epithelial cells, offering a reliable platform for comparing the expression and function of miRNA (Sabit et al. 2021). Researchers may use these models to pinpoint important miRNA targets and clarify how they contribute to the pathophysiology of breast cancer.

This study focuses on investigating the differential expression and regulatory roles of five key miRNAs: miR‐19b, miR‐20a, miR‐126, miR‐155, and miR‐200c in MDA‐MB‐231 breast cancer cells and hTERT‐HME1 healthy cells. These miRNAs were selected based on their well‐established involvement in critical pathways governing cancer progression, including cell proliferation, migration, invasion, angiogenesis, and metastasis. By exploring their modulation in response to treatment with *B. macrantha* extract, the study aims to shed light on their potential as biomarkers and therapeutic targets, particularly in aggressive breast cancer subtypes like triple‐negative breast cancer (TNBC).

The primary goal is to assess how *B. macrantha* extract influences the expression of oncogenic miRNAs (miR‐19b, miR‐20a, and miR‐155) and tumor‐suppressive miRNAs (miR‐126 and miR‐200c). Oncogenic miRNAs such as miR‐19b and miR‐20a are known to promote tumor growth and angiogenesis by targeting critical tumor suppressor genes like PTEN, while miR‐155 is implicated in inflammatory pathways driving aggressive tumor behavior. Conversely, tumor‐suppressive miRNAs like miR‐126 and miR‐200c play crucial roles in inhibiting angiogenesis and epithelial–mesenchymal transition (EMT), respectively, thereby suppressing metastasis and tumor progression.

By examining the differential effects of the extract on miRNA expression in cancerous versus healthy cells, the study seeks to highlight its selective action, offering insights into its potential therapeutic specificity. The upregulation of tumor‐suppressive miRNAs and suppression of oncogenic miRNAs may provide a balanced approach to targeting multiple pathways involved in cancer development. These findings not only suggest the therapeutic potential of *B. macrantha* but also emphasize the importance of miRNAs as precision medicine tools for diagnosing and treating breast cancer.

Moreover, the research contributes to the identification of miRNAs as biomarkers for early detection and prognostic evaluation, particularly in challenging subtypes like TNBC. By integrating miRNA modulation with plant‐based therapeutic strategies, this study lays the groundwork for advancing precision oncology and improving patient outcomes through more targeted, effective, and safer cancer treatments.

## 2 Materials and Methods

### 2.1 Collection of Plant Material

**FIGURE 2:**
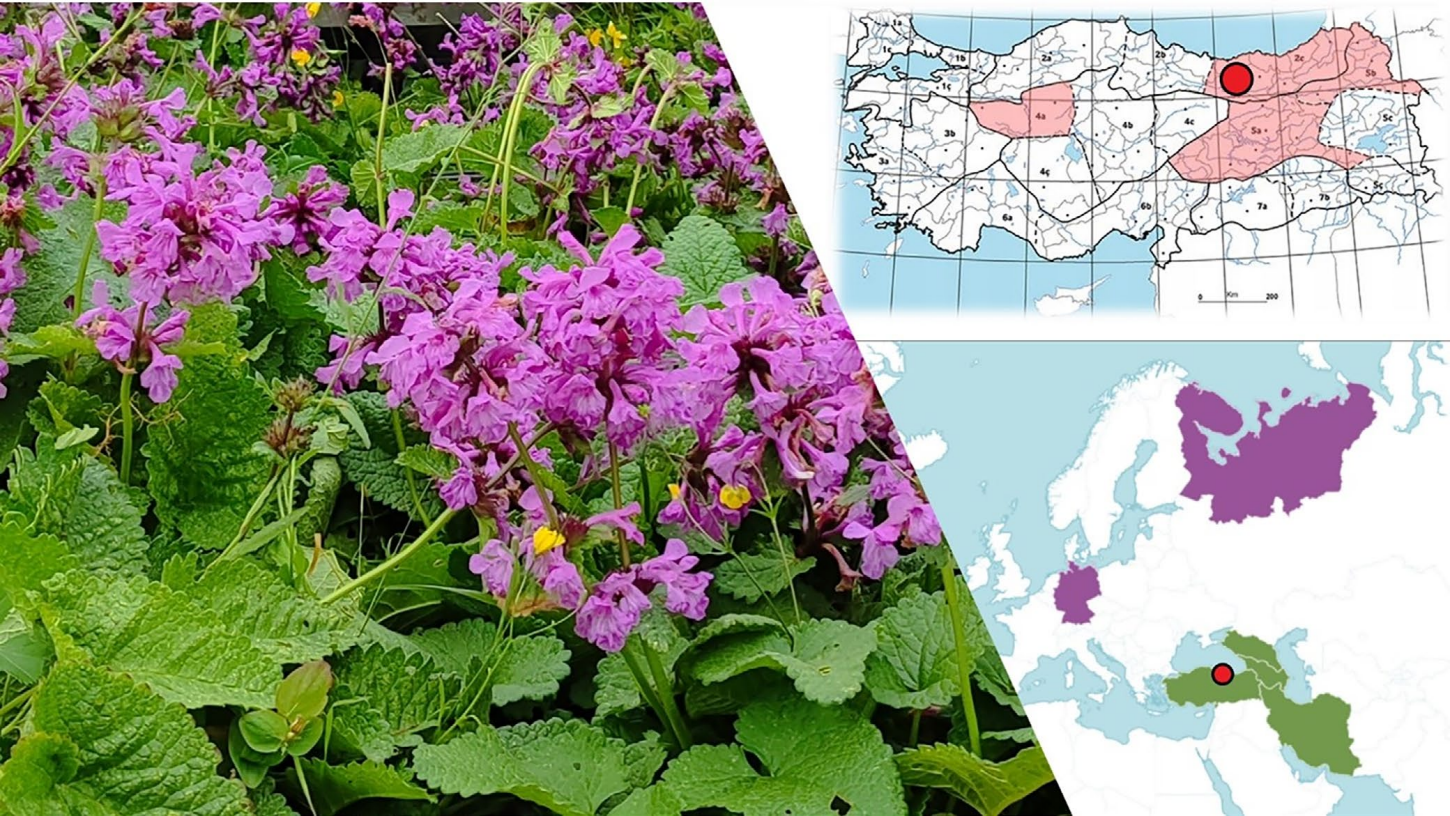
Distribution and collection sites of *Betonica macrantha*.

The above‐ground parts of *B. macrantha* were collected on July 25, 2023, from slopes with dense populations at an altitude of 2100 m in the Çambaşı Plateau region of Ordu. The specimens were identified by Dr. Alper Durmaz using the Flora of Turkey (Davis 1982) (Figure 2). Their current taxonomic status was verified using the POWO database (POWO 2024). The herbarium sample is stored at the Herbarium of the Department of Biology, Faculty of Science, Ondokuz Mayıs University, with the accession number OMUB‐4959.

### 2.2 Plant Material Extraction

The above‐ground parts of the *B. macrantha* plant were washed with distilled water and dried in the shade for 7 days. Subsequently, the above‐ground parts were dried in an oven at 40°C for 2 days and then ground into a fine powder. One hundred grams of dried sample were placed into bottles, and 1000 mL of methanol (1:10 ratio g/mL) was added. The solution was stirred occasionally and kept in the dark for 72 h. The solutions were then filtered through Whatman filter paper and evaporated using a rotary evaporator (Heidolph, Germany) at 40°C. The solid extracts were stored at 4°C and prepared for further use (Aytar and Aydın 2025).

### 2.3 Extraction and Analysis of Fatty Acids by GC–MS

A total of 5 g of the plant sample was ground into a fine powder using a laboratory grinder. The powdered sample was placed in a standard filter paper and subjected to Soxhlet extraction using an automated Soxhlet apparatus. Hexane was used as the solvent for a duration of 4 h. After the extraction, the solvent was evaporated, and the extracted oil was collected in vials for further analysis. To prepare fatty acid methyl esters, approximately 0.1 g of oil was weighed into a 5 mL glass tube. Subsequently, 2 mL of *n*‐hexane was added, and the mixture was thoroughly vortexed. To this mixture, 0.2 mL of 2 N methanolic KOH was added, and the tube was tightly sealed and vigorously mixed for 30 s. The tubes were centrifuged to achieve clear phase separation and allowed to stand for a short period. The upper phase, containing the methyl esters, was carefully transferred into vials using a Pasteur pipette (Kesen et al. 2016). The GC–MS analysis was performed using an Agilent gas chromatograph (Agilent Technologies, Santa Clara, CA, USA) equipped with an HP‐88 capillary column (60 m × 0.25 mm × 0.20 μm). High‐purity helium (> 99.99%) was used as the carrier gas at a constant flow rate of 1.0 mL/min. The GC temperature program was initiated at 140°C and held for 5 min. The temperature was then increased at a rate of 4°C/min until reaching 250°C, where it was held constant for 10 min. The injection volume was 1 μL with a split ratio of 1:50. Mass spectrometry analysis was conducted in electron ionization (EI) mode with an ionization energy of 70 eV. The ion source temperature was set to 230°C, and the mass scan range was set between *m*/*z* 30 and 550 (Zhang et al. 2022).

### 2.4 Extraction and Quantification of Phenolic Compounds by HPLC‐DAD

A dry powder sample (1 g) was extracted using 10 mL of methanol. The extraction process began with ultrasonication for 30 min to enhance the release of target compounds into the solvent. Following this step, the mixture was transferred to a shaker and incubated in darkness at room temperature for 24 h, allowing for efficient extraction under controlled conditions. After incubation, the extracts were filtered through ordinary filter paper to remove larger particles. The filtrate was then further purified using a 0.45 μm syringe filter to ensure the removal of finer particulates, resulting in a clear and purified extract ready for analysis. Chromatographic separation was performed on an ACE 5 C18 column (250 mm × 4.6 mm, 5 μm particle size) using a mobile phase composed of acetonitrile (Solvent A) and 1.5% acetic acid solution (Solvent B). A gradient elution was employed, starting with 15% Solvent A and 85% Solvent B, transitioning to 40% Solvent A and 60% Solvent B over 29 min. The HPLC system included a 1260 DAD WR detector monitoring wavelengths at 250, 270, and 320 nm, a 1260 Quat Pump with a flow rate of 0.7 mL/min, a 1260 Vialsampler injecting 10 μL of the sample, and a G7116A column oven set to 35°C. The quantification of phenolic compounds was achieved using calibration curves constructed with six standard concentrations (25, 50, 75, 100, 200, and 300 μg/mL). The extracted samples were analysed using HPLC‐DAD, ensuring precise identification and quantification. Combining ultrasonication, extended incubation, dual‐stage filtration, and chromatographic separation, this method maximized compound recovery and delivered reliable analytical results.

### 2.5 Preparation of Extract

The plant extract of *B. macrantha* was dissolved in methanol and was filtered by 0.22 μm syringe filter to obtain a stock solution of 100 mg/mL. Subsequently, working solutions (60–1250 μg/mL) were prepared from the stock solution by diluting with DMEM and used in the analysis.

### 2.6 Cell Culture

Human breast adenocarcinoma cells (MDA‐MB‐231) and non‐cancer hTERT‐immortalized mammary epithelial cells (hTERT‐HME1), used as a healthy control cell, were obtained from the American Type Culture Collection (ATCC). The cells were cultured in Dulbecco's modified Eagle's medium (DMEM) (Diagnovum, The Netherlands) supplemented with 10% fetal bovine serum (FBS) (Gibco, USA) and 1% penicillin/streptomycin (Gibco, USA) in a humidified 5% CO_2_ incubator at 37°C.

### 2.7 Cell Proliferation Assay

Cell proliferation using the MTT [3‐(4,5‐dimethylthiazol‐2‐yl)‐2,5‐diphenyltetrazolium bromide] assay was performed as described previously (Sarı et al. 2024). Briefly, MDA‐MB‐231 cells (1 × 10^4^ cells/well) were seeded in a 96‐well plate and allowed to adhere by incubating at 37°C for 24 h before applying the treatments. The cells were then exposed to varying concentrations of the extract (0.06–1.25 mg/mL) for 72 h. Due to the colored nature of the extract, which interfered with accurate absorbance readings, the contents of all wells were removed prior to adding MTT. The wells were then washed with PBS to remove residual extract. Subsequently, 120 μL of MTT solution (20 mg/mL) was added to each well, and the plates were incubated for 3 h at 37°C to allow for the formazan crystal formation. After the incubation period, the MTT solution was carefully removed, and 150 μL of dimethyl sulfoxide (DMSO) was added to each well to dissolve the formazan crystals. The absorbance was recorded at 570 nm using a microplate reader to assess cell viability. The absorbance values obtained for each concentration were normalized and expressed as a percentage of cell viability. The normalized % cell viability values and corresponding concentrations were input into the GraphPad Prism 8 data analysis software, where dose–response curves were generated. The IC_50_ value was calculated using nonlinear regression with a sigmoid curve fit method and utilized for subsequent miRNA expression analyses.

### 2.8 Expression Analysis of Target miRNAs

Real time quantitative polymerase chain reaction (RT‐qPCR) was performed to assess the impact of *B. macrantha* on the miRNA expression levels of mir‐19b, mir‐20a, mir‐126, mir‐155, mir‐200c. miRNA was extracted using the BioBasic miRNA Isolation Kit (BioBasic, Canada) according to the manufacturer's instructions. cDNA synthesis from miRNA was performed using the Poly A Polymerase Kit (ABM, Canada) and the cDNA Synthesis Kit (ABM, Canada), ensuring that all steps were conducted on ice to preserve RNA integrity. The synthesized cDNA was then used to prepare the real‐time PCR reaction mix, which was analyzed using the LightCycler 480 system (Roche, Germany) for RT‐qPCR analysis. The miRNA primers used in the qPCR were as follows: hsa‐miR‐19b Cat: MPH02295, hsa‐miR‐20a‐5p Cat: MPH02317, hsa‐miR‐126‐5p Cat: MPH02125, hsa‐miR‐155‐5p Cat: MPH02225, hsa‐miR‐200c‐3p Cat: MPH02300. mRNA fold changes were determined relative to U6, which served as the internal reference of miRNAs (Li et al. 2020). The reference gene U6 was used for normalization, and its forward primer sequence was 5′‐CTCGCTTCGGCACATA‐3′. The primer was purchased from Biomers.net with the catalog number 00180‐854. The comparative threshold cycle (ΔΔCT) method was used for the calculation. Specifically, the expression differences between the treated and untreated cancer cells were assessed using the formula 2^−(ΔΔCT)^.

### 2.9 Prediction and Analysis With admetSAR 3.0

In this research, the ADMET (Absorption, Distribution, Metabolism, Excretion, Toxicity) characteristics, pharmacokinetic behaviors, and drug‐like properties of the phytochemicals alpha‐pinene, caryophyllene, humulene, (E)‐beta‐farnesene, alpha‐cadinene, oleuropein, catechin, and rutin were analyzed using the admetSAR 3.0 platform (https://lmmd.ecust.edu.cn/admetsar3). This tool utilizes a comprehensive database and sophisticated algorithms to forecast the pharmaceutical and biological traits of chemical compounds. The evaluation included absorption‐related parameters such as solubility (logS), pKa values, Caco‐2 permeability, and human intestinal absorption (HIA). Additionally, distribution properties, including blood–brain barrier (BBB) penetration, plasma protein binding percentages (PPB), and volume of distribution (VDss), were examined. Metabolic profiling focused on the interaction of these phytochemicals with CYP450 enzymes as inhibitors and substrates, as well as their metabolic activity in human and rat liver microsomes. For excretion, parameters such as plasma clearance (CLp), renal clearance (CLr), and elimination half‐life (*T*1/2) were determined. Pharmacokinetic indicators, including molecular weight, counts of hydrogen bond acceptors and donors (HBA and HBD), and topological polar surface area (TPSA), were also analyzed alongside physicochemical properties. The compounds were assessed according to Lipinski, Pfizer, and GlaxoSmithKline (GSK) guidelines, and their drug‐like potential was quantified using the Quantitative Estimate of Drug‐likeness (QED) metric. All parameters were compiled in a table format, showing percentage success rates for each. The findings provided in‐depth insights into the pharmacokinetic compatibility and toxicity profiles of these phytochemicals (Yang et al. 2019; Cheng et al. 2012).

### 2.10 Molecular Docking Analysis

In this study, molecular docking simulations were performed to explore the interactions of alpha‐pinene, caryophyllene, humulene, (E)‐beta‐farnesene, alpha‐cadinene, oleuropein, catechin, and rutin with specific target proteins. The three‐dimensional structures of the target proteins were obtained from the Protein Data Bank (https://www.rcsb.org/), using PDB IDs 1D5R (crystal structure of the PTEN tumor suppressor), 4P7U (extracellular domain of type II transforming growth factor beta receptor in complex with NDSB‐201), 7SJ3 (structure of CDK4‐Cyclin D3 bound to abemaciclib), 8T35 (crystal structure of K51 acetylated LC3A in complex with the LIR of TP53INP2/DOR), and 3NAR (crystal structure of ZHX1 HD4). Prior to docking, water molecules and cofactors were removed from the protein structures, and polar hydrogen atoms were added using the AutoDockTools (ADT) software. Ligand structures, including alpha‐pinene, caryophyllene, humulene, (E)‐beta‐farnesene, alpha‐cadinene, oleuropein, catechin, and rutin, were retrieved from the PubChem database in SDF format and converted to PDB format using Discovery Studio Visualizer. To define the binding regions of the target proteins, the AutoGrid program was employed. The grid center was located around the active site, with dimensions of 40 points in each direction and a spacing of 0.375 Å. Docking simulations were conducted using AutoDock Vina, evaluating ten binding modes for each ligand‐protein interaction to estimate binding affinities. The energy range was set to 9 kcal/mol, and the exhaustiveness parameter was adjusted to 1000 to enhance the reliability of the results. The docking outcomes were analyzed based on binding energy, ligand efficiency (LE), fit quality (FQ), ligand lipophilic efficiency (LLE), ligand efficiency‐dependent lipophilicity (LELP), and the estimated inhibition constant (*K*_i_). The binding poses and interaction sites of the ligands with the target proteins were visualized in 2D and 3D using BIOVIA Discovery Studio Visualizer (Biovia 2021). These techniques provided detailed insights into the binding potential and pharmacological capabilities of the compounds with the selected protein targets.

### 2.11 Statistical Analysis

The data were analyzed using GraphPad Prism version 8, and the Student's *t*‐test was performed to determine the statistical significance between groups, specifically for comparing treated and untreated cell groups. Results are expressed as the mean ± standard deviation (SD). A *p*‐value less than 0.05 was considered statistically significant. Results are expressed as the mean ± standard deviation (SD).

## 3 Results and Discussion

### 3.1 GC–MS Result of *B. macrantha*

The phytochemical composition of *B. macrantha* was analyzed using GC–MS (Table 1). This analysis identified the volatile components of the plant, revealing a total of 42 distinct compounds. The identification of these compounds was based on their retention times and retention indices. The most abundant compounds were 1S‐α‐pinene (15.136%), humulene (14.671%), and caryophyllene (7.588%). Other notable compounds included beta‐myrcene (4.842%), germacrene D (3.416%), and (E)‐beta‐famesene (5.246%). These findings highlight the rich chemical profile of the plant's essential oils, suggesting its potential as a valuable source for biological activities. The table below summarizes the identified compounds, along with their retention times, retention indices, and percentages.

**TABLE 1:** Phytochemical compounds of *B. macrantha* .

No.	Retention time (min)	Retention index	Name of the compounds	Content %
1	10.919	927	3‐Thujene	1.709
2	11.229	933	1S‐α‐pinene	15.136
3	11.919	948	Camphene	1.094
4	13.032	971	α‐Sabinene	1.094
5	13.096	973	4‐Thujene	2.789
6	13.24	976	Beta‐pinene	1.422
7	13.802	988	Sulcatone	0.246
8	13.979	991	Beta‐myrcene	4.842
9	14.636	1005	Alpha‐phellandrene	0.555
10	15.262	1016	α‐Terpinene	2.007
11	15.69	1024	p‐Cymene	2.203
12	16.396	1037	Trans‐beta‐ocimene	1.306
13	16.947	1048	Ocimene	0.172
14	17.487	1058	Gamma‐terpinene	2.328
15	19.707	1099	Undecane	0.401
16	21.296	1129	Neo‐allo‐ocimene	0.340
17	32.977	1349	Alpha‐cubebene	0.236
18	34.079	1370	Ylangene	0.157
19	34.304	1375	Copaene	1.641
20	34.758	1384	Beta‐bourbonene	1.904
21	35.02	1389	İsoledene	0.205
22	35.304	1395	*cis*‐Muurola‐3,5‐diene	0.212
23	36.470	1414	Caryophyllene	7.588
24	36.925	1428	*cis*‐β‐copaene	0.512
25	37.261	1435	2‐Norpinene	0.464
26	37.406	1438	Aromandendrene	0.362
27	37.780	1446	Naphthalene	0.377
28	11.919	948	Camphene	28
29	38.139	1453	Humulene	14.671
30	38.295	1456	(E)‐beta‐famesen	5.246
31	38.568	1462	*cis*‐Muurola‐4(15), 5‐diene	0.734
32	39.235	1476	Gamma‐muurolene	1.558
33	39.444	1480	Germacrene D	3.416
34	40.177	1495	β‐Cyclogermacrane	0.360
35	40.289	1497	Epizonarene	1.102
36	40.353	1499	α‐Muurolene	1.334
37	40.744	1507	Beta‐bisabolene	1.334
38	41.0	1512	γ‐Cadinene	0.620
39	41.435	1520	*cis*‐Calamenene	2.362
40	42.089	1533	α‐Cadinene	5.802
41	42.332	1538	Alpha‐calacorene	0.342
42	49.403	1678	Cadalene	0.088

### 3.2 HPLC‐DAD Result of *B. macrantha*

The bioactive compounds in the *B. macrantha* extract were measured using HPLC, and a total of 18 different bioactive compounds were identified (Table 2). The most abundant compounds were catechin (3265.35 mg/L), oleuropein (1955.69 mg/L), and rutin (1734.7 mg/L). Additionally, significant amounts of ascorbic acid (966 mg/L), gallic acid (181.51 mg/L), and ferulic acid (144.86 mg/L) were detected. The extract exhibited a rich profile of phenolic acids, flavonoids, and other bioactive compounds, highlighting its potential as a valuable natural source for pharmacological and biological activities. The table below presents the identified bioactive compounds and their concentrations (mg/L).

**TABLE 2:** Bioactive compounds in *B. macrantha* extract measured by HPLC.

No.	Compounds	(mg/L)
1	Ascorbic acid	966
2	Gallic acid	181.51
3	4‐Hydroxybenzoic acid	3.11
4	Vanillic acid	7.57
5	Coumarıc Acid	77.85
6	Caffeic acid	31.93
7	Ferulic acid	144.86
8	Rosmarinic acid	15.62
9	Pyrogallol	14.44
10	Chlorogenic Acid	153
11	Resveratrol	77.19
12	Oleuropein	1955.69
13	Catechin	3265.35
14	Epicatechin	20.64
15	Rutin	1734.7
16	Myricetin	85.73
17	Quercetin	37.68
18	Baicalin	15.28

### 3.3 The Extract of *B. macrantha* Inhibits Cell Viability in MDA Cells in a Dose‐Dependent Manner

**FIGURE 3:**
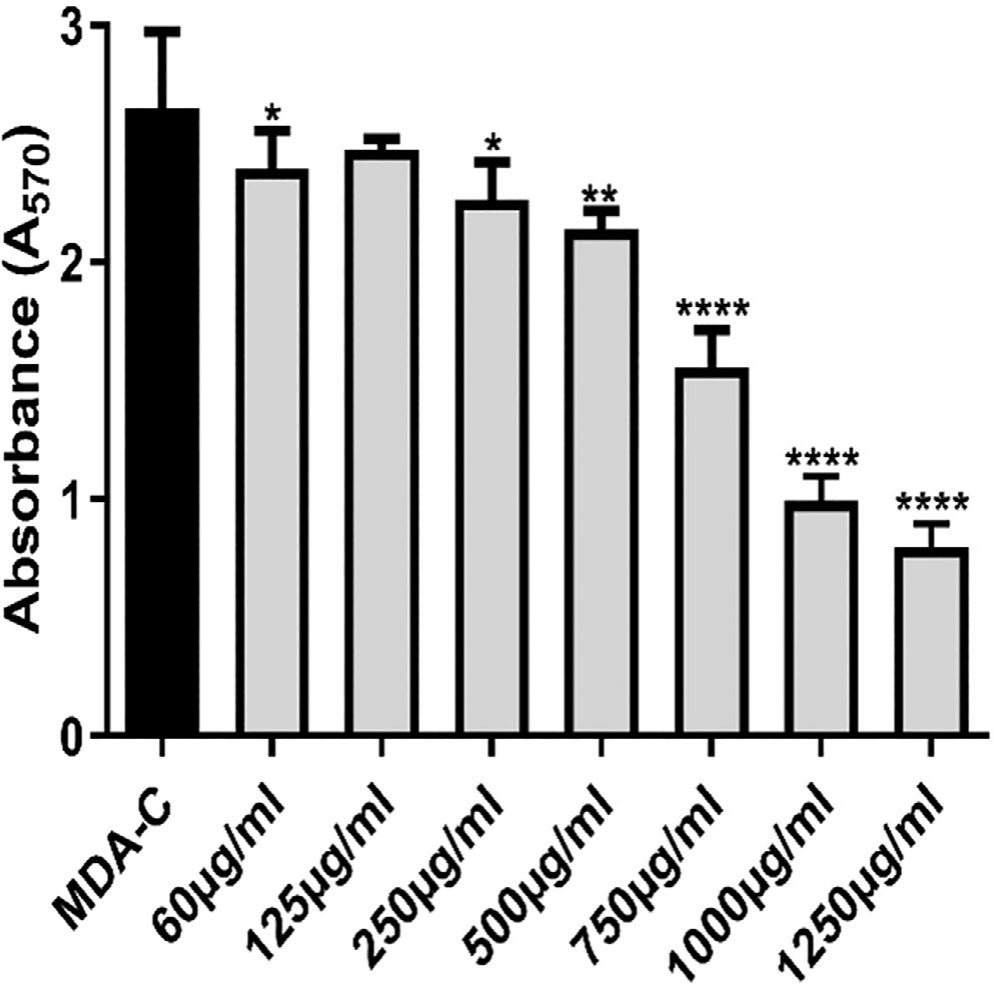
Inhibition of cell proliferation by *B. macrantha* . The effect of *B. macrantha* extract on MDA cell viability was assessed using the MTT assay. The extract significantly reduced cell viability in a dose‐dependent manner. Student's *t*‐test was performed between groups for comparing treated and untreated cell groups. Statistical significance is indicated as *p < 0.05, **p < 0.0,1, ****p < 0.0001. MDA‐C represents the untreated control group for MDA‐MB‐231 cells.

According to the MTT assay result, the proliferation of MDA cells was inhibited after treatment with different concentrations of *B. macrantha* for 72 h (Figure 3). The IC_50_ value for MDA‐MB‐231 cells treated with the extract for 72 h was determined to be 0.8 mg/mL. This concentration was subsequently applied to both cancer MDA‐MB‐231 cells and control hTERT‐HME1 cells for 72 h to investigate its effect on miRNA expression. The miRNA expression levels were analyzed to compare the responses of cancerous and healthy cells to the extract treatment.

### 3.4 The Regulatory Effects of *B. macrantha* Extract on miRNA Expression in Cancerous and Control Cells

**FIGURE 4:**
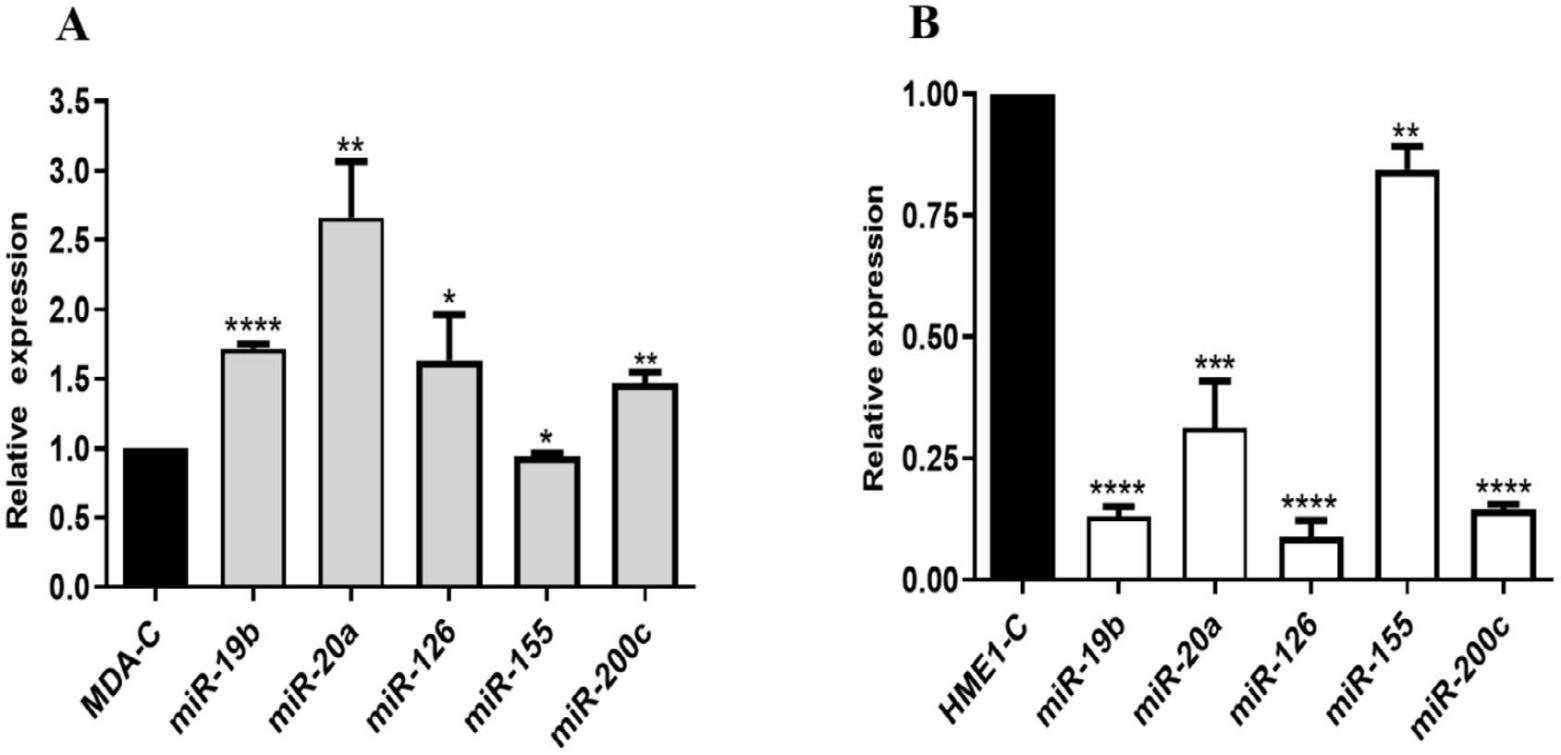
The effect of *B. macrantha* on miRNA expression in cancer and normal cells. Relative expression levels of miR‐19b, miR‐20a, miR‐126, miR‐155, and miR‐200c miRNAs in (A) MDA‐MB‐231 and (B) hTERT‐HME1 cell lines calculated using the 2^(−ΔΔCt)^ method normalized to the U6 reference gene. The results are presented as mean ± standard deviation (SD) from three independent experiments. Student's *t*‐test was performed between groups for comparing treated and untreated cell groups. Statistical significance is indicated as *p < 0.05, **p < 0.01, ***p < 0.001, ****p < 0.0001. (MDA‐C represents the untreated control group for MDA‐MB‐231 cells. HME1‐C represents the untreated control group for hTERT‐HME1 cells).

The extract treatment significantly influenced miRNA expression in MDA‐MB‐231 cancer cells, leading to notable changes in the levels of the miRNAs (Figure 4A). miR‐19 expression showed a significant 1.7‐fold increase compared to the control group (*p* < 0.0001), suggesting its upregulation in response to the treatment, while miR‐20a exhibited the highest increase among the evaluated miRNAs with a 2.6‐fold upregulation (*p* < 0.01), indicating its potential role as a prominent target of the extract. Similarly, miR‐126 demonstrated a statistically significant 1.6‐fold increase in expression (*p* < 0.05), and miR‐200c showed a 1.4‐fold increase with statistical significance (*p* < 0.01). In contrast, miR‐155 exhibited a slight decrease in expression, with a fold change of 0.9 (*p* < 0.05), suggesting minimal effect on this miRNA.

The extract treatment resulted in a statistically significant decrease in the expression of all analyzed miRNAs in hTERT‐HME1 cells compared to the untreated group, in contrast to the upregulated miRNA expressions observed in MDA‐MB‐231 cancer cells. This opposing pattern highlights the differential regulatory effects of the extract on healthy control cells versus cancerous cells (Figure 4B). Among the analyzed miRNAs, the highest downregulation was observed in miR‐126 (*p* < 0.0001), while the lowest downregulation was noted in miR‐155 (*p* < 0.01).

### 3.5 Result of admetSAR3

**FIGURE 5:**
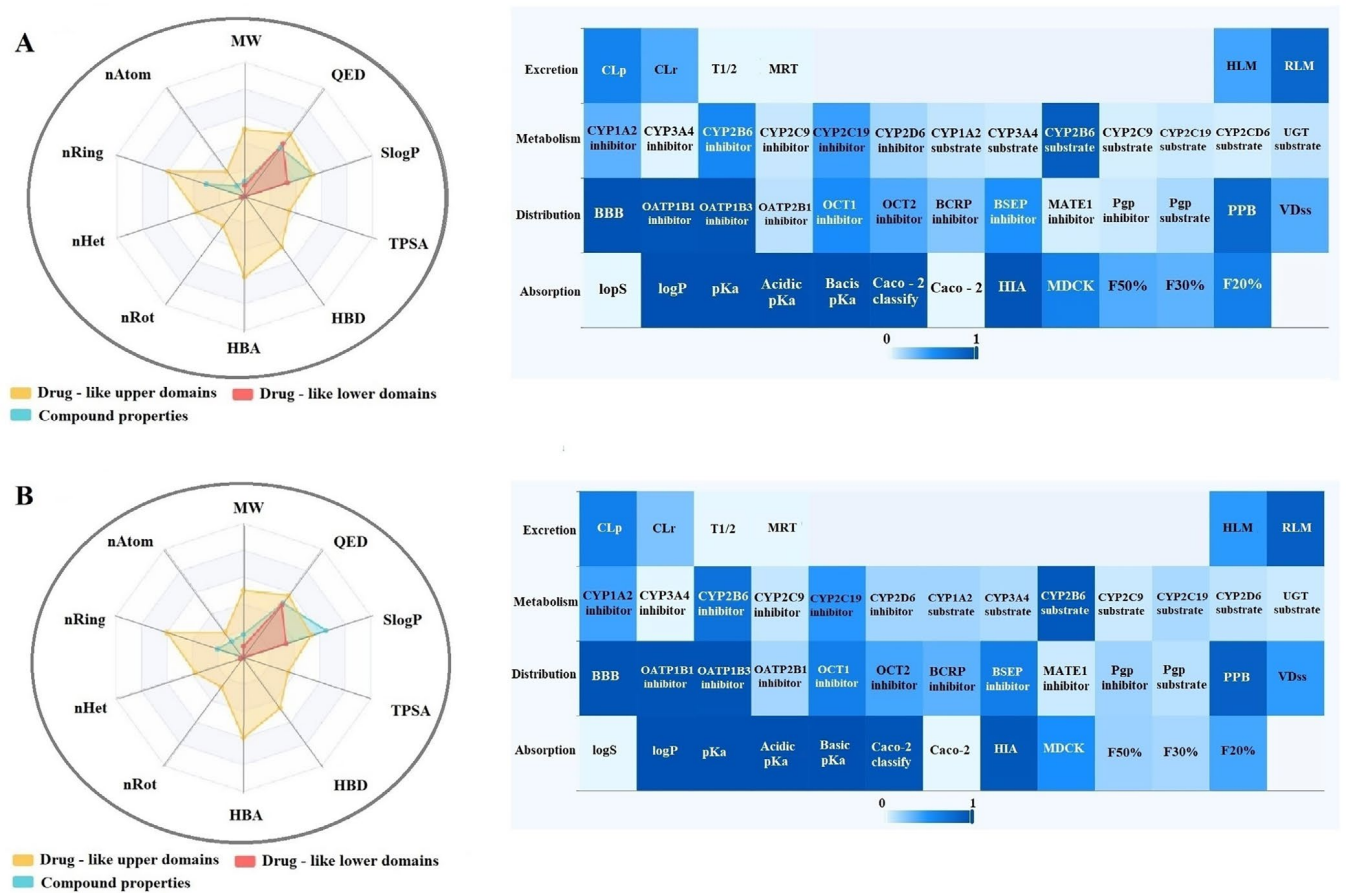
ADMET distribution properties of (A) α‐pinene and (B) caryophyllene.

**FIGURE 6:**
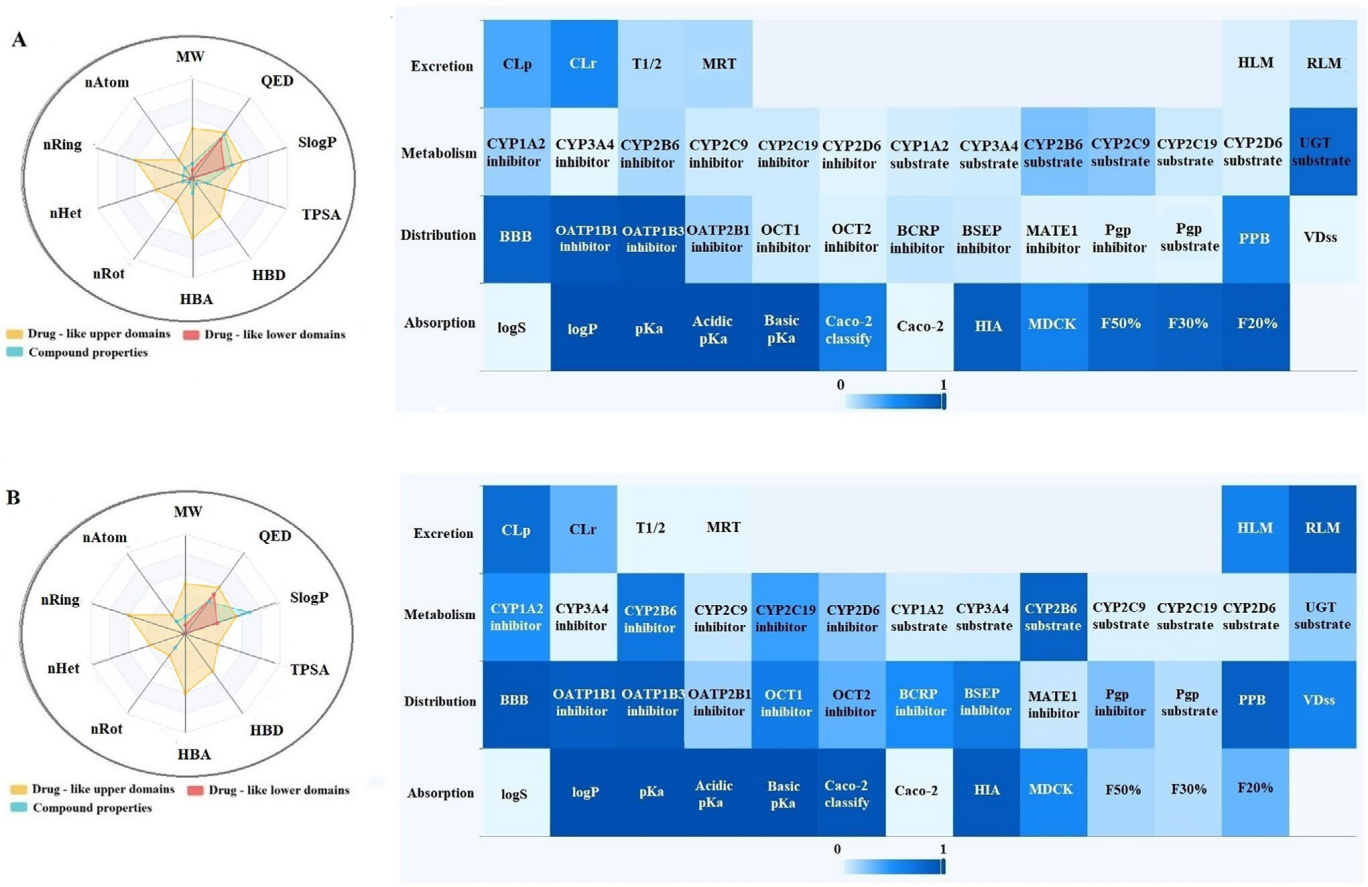
ADMET distribution properties of (A) humulene and (B) (E)‐beta‐farnesene.

**FIGURE 7:**
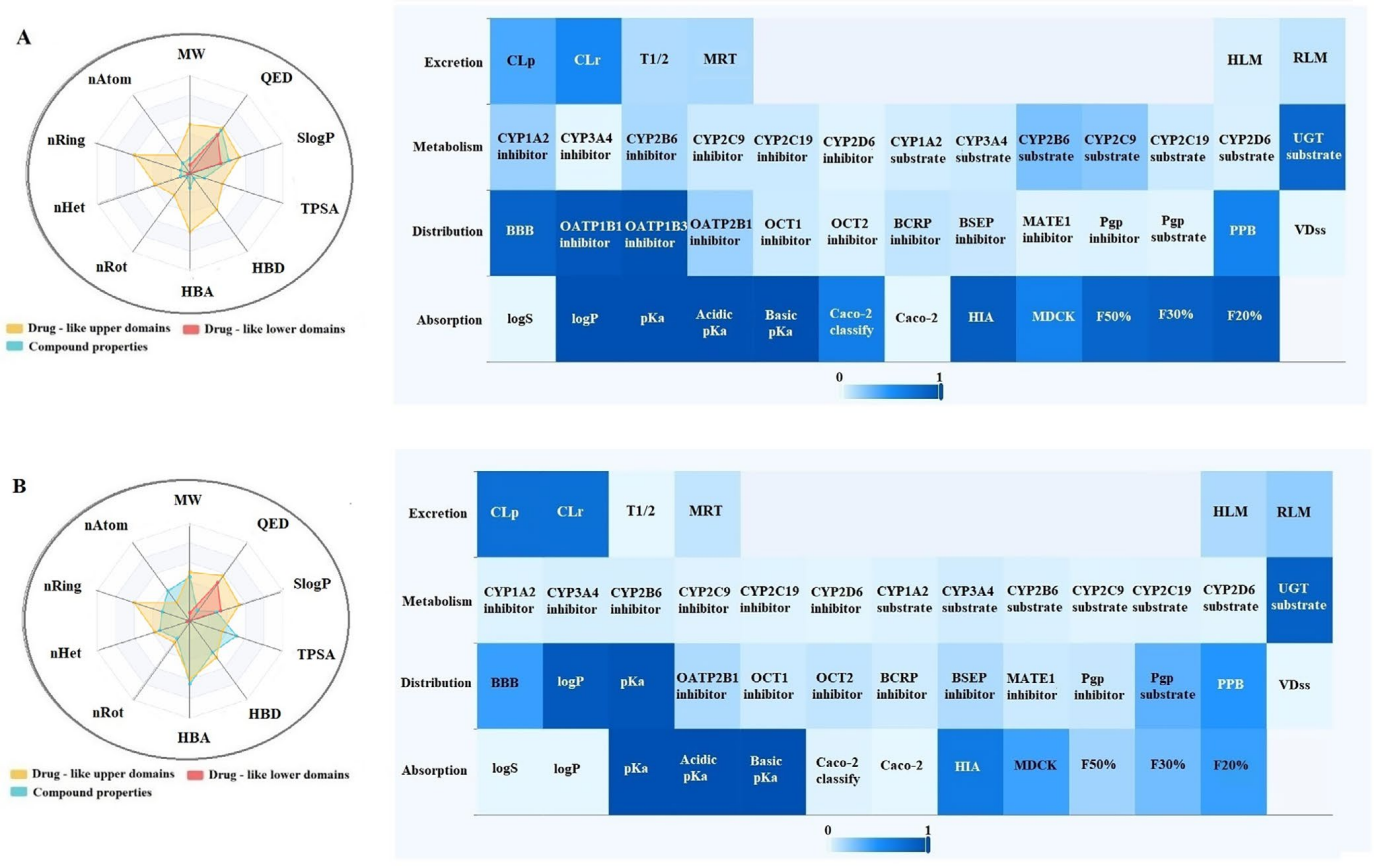
ADMET distribution properties of (A) α‐cadinene and (B) oleuropein.

**FIGURE 8:**
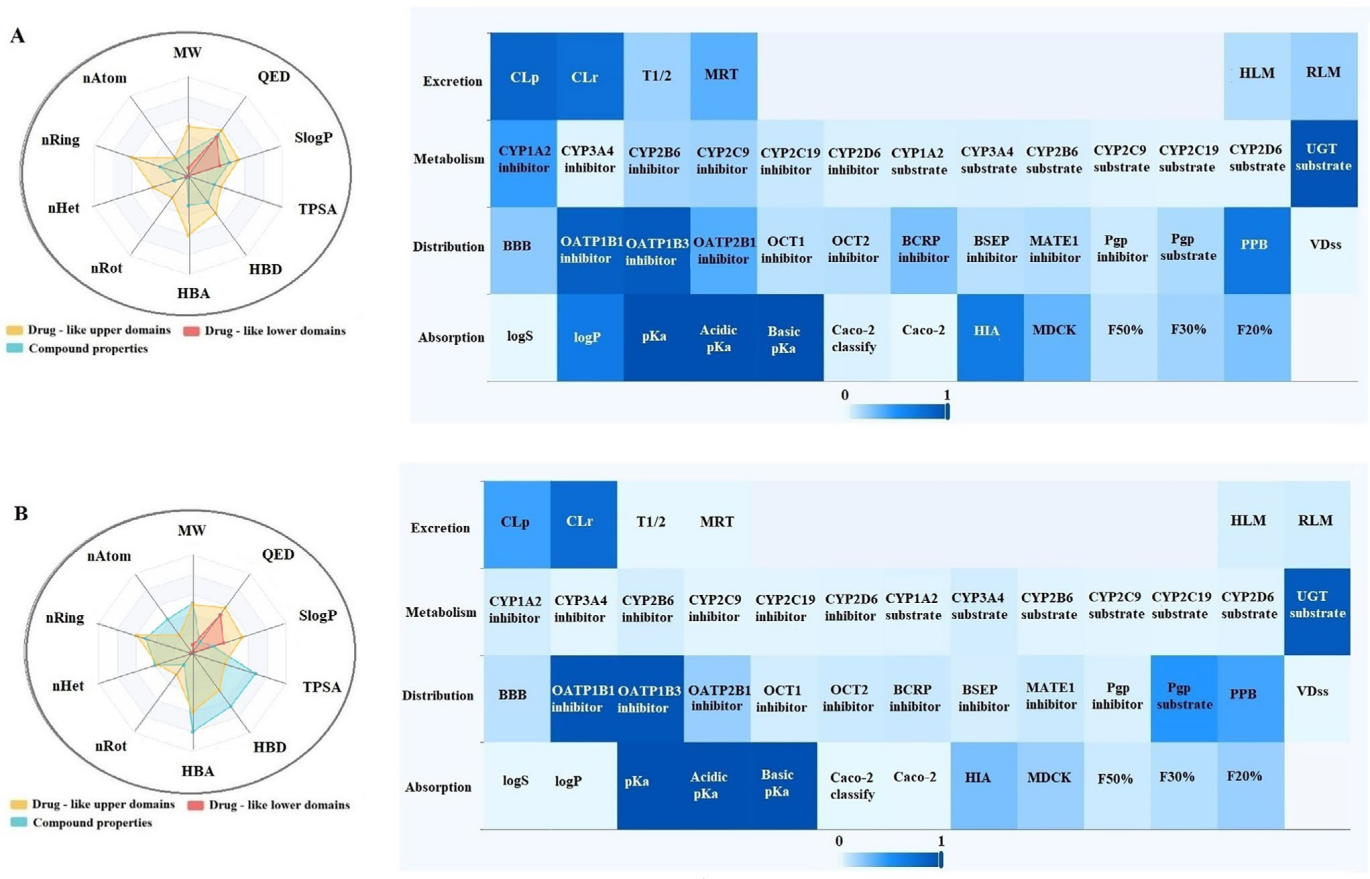
ADMET distribution properties of (A) catechin and (B) rutin.

This study comprehensively analyzed the physicochemical properties and drug‐likeness of the phytochemicals alpha‐pinene, caryophyllene, humulene, (E)‐beta‐farnesene, alpha‐cadinene, oleuropein, catechin, and rutin (Figures 5, 6, 7, 8). The evaluation included parameters such as molecular weight, number of atoms (nAtom), number of heteroatoms (nHet), number of rings (nRing), number of rotatable bonds (nRot), hydrogen bond acceptors (HBA) and donors (HBD), topological polar surface area (TPSA), and logarithmic partition coefficient (SlogP) (Table 3). Among the compounds, alpha‐pinene exhibited the lowest molecular weight (136.24 g/mol), while rutin had the highest (610.52 g/mol). The number of rotatable bonds, an indicator of structural flexibility, was highest for (E)‐beta‐farnesene with seven rotatable bonds, while alpha‐pinene and caryophyllene lacked any rotatable bonds. Oleuropein (13 HBA, 6 HBD) and rutin (16 HBA, 10 HBD) had the highest hydrogen bond acceptor and donor counts, suggesting significant polar interactions. TPSA, a critical parameter for bioavailability, was notably high for oleuropein (201.67 Å^2^) and rutin (269.43 Å^2^) but was zero for alpha‐pinene and caryophyllene, reflecting their hydrophobic nature. In terms of lipophilicity, alpha‐pinene, humulene, and catechin exhibited moderate SlogP values of 1.31. Caryophyllene (4.73) and (E)‐beta‐farnesene (5.20) demonstrated high lipophilicity, potentially indicating challenges in solubility. Conversely, oleuropein (−0.63) and rutin (−1.69) were characterized as hydrophilic, suggesting better water solubility but potentially reduced permeability. Drug‐likeness was assessed using QED (quantitative estimate of drug‐likeness) scores and compliance with Lipinski, Pfizer, and GlaxoSmithKline (GSK) rules. Humulene and catechin had the highest QED scores (57.14%), indicating strong drug‐likeness potential. In contrast, oleuropein (7.26%) and rutin (8.33%) exhibited low QED values, reflecting limited drug‐like characteristics. According to Lipinski's rule, alpha‐pinene, caryophyllene, humulene, (E)‐beta‐farnesene, alpha‐cadinene, and catechin were fully compliant (100%), while oleuropein and rutin did not meet the criteria due to high molecular weight and TPSA values. The Pfizer rule identified caryophyllene and (E)‐beta‐farnesene as non‐compliant (0%) due to their high lipophilicity, whereas other compounds fully adhered to this rule. Similarly, the GSK rule deemed oleuropein, rutin, and alpha‐cadinene non‐compliant, while the remaining compounds met the criteria. In conclusion, alpha‐pinene, humulene, and catechin demonstrated favorable physicochemical properties, moderate TPSA values, and compliance with drug‐likeness rules, making them promising candidates for pharmaceutical applications. Caryophyllene and (E)‐beta‐farnesene, with high lipophilicity, may present toxicity risks, while oleuropein and rutin, characterized by high polar surface area and hydrophilicity, might face limitations in bioavailability. These findings provide valuable insights into the pharmacokinetic profiles and pharmaceutical applicability of these compounds.

**TABLE 3:** Physicochemical properties of phytochemicals.

ADMET	Physicochemical property															
	Alpha‐pinene		Caryophyllene		Humulene		(E)‐beta‐farnesene		Alpha‐cadinene		Oleuropein		Catechin		Rutin	
Molecular weight	136.24		204.36		180.16		204.36		180.16		540.52		180.16		610.52	
nAtom	10		15		13		15		13		38		13		43	
nHet	0		0		4		0		4		13		4		16	
nRing	3		2		1		0		1		3		1		5	
nRot	0		0		2		7		2		9		2		6	
HBA (hydrogen bond acceptor)	0		0		3		0		3		13		3		16	
HBD (hydrogen bond donor)	0		0		1		0		1		6		1		10	
TPSA (topological polar surface area)	0		0		63.60		0		63.60		201.67		63.60		269.43	
SlogP (logarithmic partition coefficient)	1.31		4.73		1.31		5.20		1.31		−0.63		1.31		−1.69	
Medicinal chemistry																
QED (quantitative estimate of drug‐likeness)	0.45	45.11%	0.50	51.30%	0.55	57.14%	0.40	38.69%	0.55	57.14%	0.13	7.26%	0.55	57.14%	0.14	8.33%
Lipinski rule	Accept	100%	Accept	100%	Accept	100%	Accept	100%	Accept	100%	Not accept	0%	Accept	100%	Not accept	0%
Pfizer rule	Accept	100%	Not accept	0%	Accept	100%	Not accept	0%	Accept	100%	Accept	100%	Accept	100%	Accept	100%
GSK rule (GlaxoSmithKline rule)	Accept	100%	Not accept	0%	Accept	100%	Not accept	0%	Accept	100%	Not accept	0%	Accept	100%	Not accept	0%

In this study, the ADME (Absorption, Distribution, Metabolism, and Excretion) properties of alpha‐pinene, caryophyllene, humulene, (E)‐beta‐farnesene, alpha‐cadinene, oleuropein, catechin, and rutin were evaluated (Table 4). According to the absorption parameters, solubility (logS) values indicated that humulene (−1.51) and catechin (−1.51) exhibited the highest solubility, whereas caryophyllene (−5.55) and (E)‐beta‐farnesene (−5.29) showed lower solubility levels. Lipophilicity analysis revealed that caryophyllene (84.72%) and (E)‐beta‐farnesene (82.33%) had high logP values, reflecting their lipophilic nature. Conversely, oleuropein (−0.15) and Rutin (−1.14) showed low logP values, indicating hydrophilic properties. Caco‐2 permeability results demonstrated that alpha‐pinene (97.10%) and caryophyllene (96.90%) had high permeability across the intestinal epithelium, while oleuropein (1.80%) and rutin (0.20%) showed minimal permeability. Human intestinal absorption (HIA) results showed that alpha‐pinene (90.50%), caryophyllene (93.90%), and humulene (90.60%) had high absorption potential, whereas oleuropein (68.00%) and rutin (26.70%) exhibited limited absorption. For distribution parameters, blood–brain barrier (BBB) permeability results revealed that alpha‐pinene (98.40%) and caryophyllene (97.90%) had a high potential to penetrate the central nervous system. In contrast, oleuropein (42.70%) and rutin (10.70%) exhibited poor BBB permeability. Plasma protein binding (PPB) analysis indicated that caryophyllene (87.40%) and (E)‐beta‐farnesene (87.70%) had a high binding affinity to plasma proteins, while oleuropein (47.60%) and rutin (40.10%) showed lower binding levels. Volume of distribution at steady state (VDss) results revealed that humulene and catechin (15.34%) had limited tissue distribution, while caryophyllene (56.20%) and (E)‐beta‐farnesene (61.87%) displayed broader distribution profiles. In conclusion, alpha‐pinene and caryophyllene demonstrated favorable pharmacokinetic properties, including high absorption, permeability, and distribution, making them promising candidates for pharmaceutical applications. However, their high lipophilicity may pose a potential toxicity risk. Humulene and catechin exhibited a balanced pharmacokinetic profile with good solubility and moderate lipophilicity. On the other hand, oleuropein and rutin showed limited absorption, low permeability, and poor plasma protein binding, which may restrict their bioavailability. These findings provide valuable insights into the pharmacokinetic profiles and pharmaceutical potential of these compounds.

**TABLE 4:** Absorption and distribution properties of phytochemicals based on ADME predictions.

ADMET	Alpha‐pinene		Caryophyllene		Humulene		(E)‐beta‐farnesene		Alpha‐cadinene		Oleuropein		Catechin		Rutin	
ADME property prediction (absorption)																
logS	−4.57	73.57%	−5.55	31.68%	−1.51	73.57%	−5.29	34.34%	1.51	73.57%	−3.00	58.11%	1.51	73.57%	−2.59	62.36%
logP	4.78	73.11%	5.86	84.72%	1.67	39.77%	5.64	82.33%	1.67	39.77%	−0.15	20.24%	1.67	39.77%	−1.14	9.59%
pKa	8.37	67.06%	10.30	80.73%	3.48	35.52%	9.47	74.86%	3.48	32.52%	6.99	57.34%	3.48	32.52%	6.89	56.60%
Acidic pKa	10.85	75.84%	11.40	79.83%	3.47	22.38%	10.04	69.99%	3.47	22.38%	6.85	46.86%	3.47	22.38%	6.95	47.58%
Basic pKa	7.92	71.89%	9.15	82.58%	2.48	33.58%	8.92	78.99%	2.48	33.58%	6.29	60.43%	2.48	33.58%	6.61	62.66%
Caco‐2	1	97.10%	1	96.90%	1	64.00%	1	95.60%	1	64.00%	0	1.80%	1	64.00%	0	0.20%
Caco‐2 (classify)	−3.93	100.00%	−4.04	100.00%	−4.61	84.60%	−3.92	100.00%	4.61	84.60%	−6.47	29.91%	4.61	84.60%	−7.22	7.58%
HIA (human intestinal absorption)	1	90.50%	1	93.90%	1	90.60%	1	93.10%	1	90.60%	1	68.00%	1	90.60%	0	26.70%
MDCK (Madin‐Darby canine kidney)	1	62.10%	1	50.90%	1	61.50%	1	56.10%	1	61.50%	0	41.30%	1	61.50%	0	18.70%
F50% (fraction of the compound)	0	36.40%	0	18.30%	1	83.80%	0	15.40%	1	83.80%	0	16.90%	1	83.80%	0	6.30%
F30%	0	33.10%	0	15.40%	1	87.40%	0	12.50%	1	87.40%	0	28.10%	1	87.40%	0	11.80%
F20%	1	63.10%	0	39.50%	1	93.00%	0	31.40%	1	93.00%	0	42.20%	1	93.00%	0	18.70%
ADME property prediction (distribution)																
BBB (blood–brain barrier)	1	98.40%	1	97.90%	1	85.00%	1	95.80%	1	85.00%	0	42.70%	1	85.00%	0	10.70%
OATP1B1 inhibitor	1	97.00%	1	95.80%	1	94.30%	1	89.30%	1	94.30%	1	92.70%	1	94.30%	1	94.70%
OATP1B3 inhibitor	1	97.70%	1	96.40%	1	98.40%	1	92.10%	1	98.40%	1	96.40%	1	98.40%	1	97.00%
OATP2B1 inhibitor	0	10.50%	0	16.00%	0	17.90%	0	19.30%	0	17.90%	0	13.90%	0	17.90%	0	19.40%
OCT1 inhibitor	1	53.00%	1	63.10%	0	6.00%	1	65.00%	0	6.00%	0	6.20%	0	6.00%	0	4.80%
OCT2 inhibitor	0	37.70%	0	39.50%	0	3.00%	0	33.60%	0	3.00%	0	8.69%	0	3.00%	0	6.30%
BCRP inhibitor	0	24.10%	0	33.40%	0	8.90%	1	52.10%	0	8.90%	0	5.80%	0	8.90%	0	7.30%
BSEP inhibitor	0	47.40%	1	64.20%	0	7.09%	1	68.30%	0	7.09%	0	13.60%	0	7.09%	0	4.40%
MATE1 inhibitor	0	4.29%	0	6.20%	0	1.80%	0	7.70%	0	1.80%	0	6.40%	0	1.80%	0	7.00%
Pgp inhibitor	0	6.80%	0	17.29%	0	2.30%	0	25.50%	0	2.30%	0	7.80%	0	2.30%	0	3.10%
Pgp substrate	0	14.70%	0	11.20%	0	0.90%	0	12.40%	0	0.90%	0	33.60%	0	0.90%	0	47.30%
PPB (plasma protein binding)	1	81.00%	1	87.40%	1	59.90%	1	87.70%	1	59.90%	0	47.60%	1	59.90%	0	40.10%
VDss (volume of distribution at steady state)	0.36	53.03%	0.45	56.20%	0.69	15.34%	0.60	61.87%	0.69	15.34%	0.15	34.58%	0.69	15.34%	0269	29.45%

This study analyzed the metabolism and excretion properties of alpha‐pinene, caryophyllene, humulene, (E)‐beta‐farnesene, alpha‐cadinene, oleuropein, catechin, and rutin. Interactions with cytochrome P450 (CYP450) enzymes were assessed for both inhibitory and substrate potentials (Table 5). The inhibition analysis revealed that all compounds showed low inhibitory potential for CYP1A2, CYP3A4, CYP2C9, and CYP2D6 enzymes, with slightly higher activity observed for humulene (14.70%) and Catechin (18.70%). For CYP2B6, alpha‐pinene (56.20%) and caryophyllene (73.10%) demonstrated significant inhibitory potential. Similarly, in the CYP2B6 substrate analysis, alpha‐pinene (90.60%) and caryophyllene (93.60%) exhibited strong interaction potential, while the other enzymes showed lower substrate activity for the tested compounds. Liver microsomal metabolism was evaluated using human liver microsomes (HLM) and rat liver microsomes (RLM). Rutin (91.80%) and catechin (86.60%) demonstrated strong interaction as substrates for UDP‐glucuronosyltransferase (UGT). In contrast, alpha‐pinene and caryophyllene showed minimal interaction with this enzyme. The HLM analysis indicated that (E)‐beta‐farnesene (59.80%) exhibited the highest metabolic potential. In the RLM analysis, alpha‐pinene (82.00%) and caryophyllene (87.90%) displayed high metabolic activity, whereas other compounds showed lower interaction levels. Excretion parameters revealed that catechin (85.00%) and rutin (75.80%) had high plasma clearance rates (CLp), while humulene (38.10%) and oleuropein (38.10%) showed lower clearance. For renal clearance (CLr), rutin (94.30%) and catechin (78.50%) demonstrated the highest excretion levels. In terms of elimination half‐life (*T*1/2), catechin (98.40%) and humulene (77.37%) had longer half‐lives, indicating extended metabolic stability. Mean residence time (MRT) analysis showed that alpha‐pinene and caryophyllene had lower values (17.90% and 23.02%), suggesting faster systemic clearance. The metabolic evaluation highlighted that alpha‐pinene and caryophyllene exhibited significant interactions with the CYP2B6 enzyme, indicating high metabolic activity. Catechin and rutin stood out with high renal clearance and long half‐lives, making them promising candidates with effective excretion profiles. Conversely, oleuropein and humulene demonstrated limited metabolic and excretion potential. These findings provide valuable insights into the pharmacokinetics and pharmaceutical applicability of the compounds. However, further investigation into the potential toxicity risks of alpha‐pinene and caryophyllene, owing to their high metabolic activity, is recommended.

**TABLE 5:** Metabolism and excretion profiles of phytochemicals based on ADME predictions.

ADMET	Alpha‐pinene		Caryophyllene		Humulene		(E)‐beta‐farnesene		Alpha‐cadinene		Oleuropein		Catechin		Rutin	
ADME property prediction (metabolism)																
CYP1A2 inhibitor	0	33.60%	0	40.69%	0	18.70%	0	49.60%	0	18.70%	0	2.10%	0	18.70%	0	5.00%
CYP3A4 inhibitor	0	1.50%	0	0.60%	0	0.40%	0	1.40%	0	0.40%	0	4.10%	0	0.40%	0	0.80%
CYP2B6 inhibitor	1	56.20%	1	73.10%	0	14.70%	1	70.30%	0	14.70%	0	4.10%	0	14.70%	0	4.40%
CYP2C9 inhibitor	0	7.60%	0	8.20%	0	6.60%	0	8.20%	0	6.60%	0	1.90%	0	6.60%	0	1.00%
CYP2C19 inhibitor	0	42.80%	0	46.40%	0	6.60%	0	42.50%	0	6.60%	0	1.50%	0	6.60%	0	0.90%
CYP2D6 inhibitor	0	16.20%	0	16.80%	0	1.50%	0	22.70%	0	1.50v	0	0.80%	0	1.50%	0	1.40%
CYP1A2 substrate	0	8.69%	0	16.70%	0	4.90%	0	5.00%	0	4.90%	0	2.20%	0	4.90%	0	5.50%
CYP3A4 substrate	0	6.60%	0	13.80%	0	6.00%	0	5.80%	0	6.00%	0	5.20%	0	6.00%	0	4.80%
CYP2B6 substrate	1	90.60%	1	93.60%	0	26.30%	1	86.00%	0	26.30%	0	3.40%	0	26.30%	0	1.50%
CYP2C9 substrate	0	4.00%	0	6.90%	0	22.80%	0	2.90%	0	22.80%	0	1.50%	0	22.80%	0	1.00%
CYP2C19 substrate	0	6.50%	0	14.50%	0	6.30%	0	3.00%	0	6.30%	0	1.80%	0	6.30%	0	0.80%
CYP2D6 substrate	0	4.40%	0	6.90%	0	3.10%	0	3.30%	0	3.10%	0	1.10%	0	3.10%	0	1.60%
HLM (human liver microsomes)	0	40.00%	0	45.30%	0	3.70%	1	59.80%	0	3.70%	0	14.40%	0	3.70%	0	4.10%
RLM (rat liver microsomes)	1	82.00%	1	87.90%	1	8.69%	1	89.60%	0	8.69%	0	20.60%	0	8.69%	0	5.40%
UGT substrate (uridine 5′‐diphospho‐glucuronosyltransferase)	0	9.30%	0	6.30%	1	81.69%	0	20.60%	1	81.69%	1	86.60%	1	81.69%	1	91.80%
ADME property prediction (excretion)																
CLp (plasma clearance)	1	60.20%	1	62.30%	0	38.10%	1	77.00%	0	38.10v	1	75.80%	1	85.00%	0	41.30%
CLr (renal clearance)	0	37.00%	0	25.40%	1	56.10%	0	32.30%	1	56.10%	1	75.20%	1	94.30%	1	78.50%
*T*1/2 (half‐life)	−1.46	26.21%	−1.59	22.08%	0.13	77.37%	−1.34	30.19%	0.13	77.37%	−0.07	70.96%	1	98.40%	−0.38	60.83%
MRT (mean residence time)	−1.42	17.90%	−1.57	23.02%	0.15	74.09%	−1.27	32.04%	0.15	74.09v	0.09	72.34v	0	17.90%	−0.32	60.26%

### 3.6 Result of Molecular Docking

This study assessed the binding interactions of alpha‐pinene, caryophyllene, humulene, (E)‐beta‐farnesene, alpha‐cadinene, oleuropein, catechin, and rutin with the target proteins 1D5R, 4P7U, 7SJ3, 8T35, and 3NAR using molecular docking simulations (Table 6).

**TABLE 6:** Results of binding interactions of the compounds with target proteins.

		Binding energy (kcal/mol)	Ligand efficiency	Fit quality (FQ)	Ligand lipophilic efficiency (LLE)	Ligand efficiency‐dependent lipophilicity (LELP)	Estimated inhibition constant (*K*_i_) (μM)	pIC_50_
Alpha‐pinene	1D5R	−5.7	0.219	0.481	1.192	21.823	51.900	4.071
	4P7U	−5.9	0.227	0.498	1.236	21.080	50.200	4.214
	7SJ3	−7.0	0.269	0.591	1.456	17.756	1.980	5.000
	8T35	−5.1	0.196	0.430	1.067	24.380	18.200	3.643
	3NAR	−5.4	0.208	0.456	1.137	23.001	12.200	3.857
Caryophyllene	1D5R	−6.7	0.172	0.562	1.144	34.100	19.800	4.790
	4P7U	−7.1	0.182	0.596	1.213	32.200	6.140	5.070
	7SJ3	−7.7	0.197	0.645	1.315	29.700	2.260	5.500
	8T35	−5.7	0.146	0.478	0.974	40.100	20.200	4.070
	3NAR	−6.0	0.154	0.502	1.025	38.100	40.400	4.290
Humulene	1D5R	−7.7	0.197	0.645	4.610	8.474	2.260	5.500
	4P7U	−7.5	0.192	0.629	4.493	8.687	2.300	5.357
	7SJ3	−8.8	0.226	0.737	5.271	7.403	0.144	6.290
	8T35	−6.6	0.169	0.553	3.948	9.874	14.300	4.714
	3NAR	−7.1	0.182	0.596	4.254	9.170	6.140	5.071
(E)‐beta‐farnesene	1D5R	−6.1	0.156	0.511	1.082	36.050	20.400	4.360
	4P7U	−5.9	0.151	0.494	1.045	37.290	51.900	4.210
	7SJ3	−6.7	0.172	0.562	1.188	32.850	19.800	4.790
	8T35	−6.1	0.156	0.511	1.082	36.050	20.400	4.360
	3NAR	−4.8	0.123	0.402	0.850	45.800	393.300	3.430
Alpha‐cadinene	1D5R	−7.1	0.182	0.596	4.250	9.170	6.140	5.071
	4P7U	−6.6	0.169	0.553	3.950	9.870	14.300	4.714
	7SJ3	−8.8	0.226	0.737	5.270	7.400	0.144	6.290
	8T35	−6.5	0.167	0.544	3.890	10.020	4.540	4.643
	3NAR	−5.3	0.136	0.402	3.170	12.300	146.000	3.793
Oleuropein	1D5R	−8.0	0.140	0.605	−53.330	−1.070	0.576	5.710
	4P7U	−6.2	0.109	0.469	−41.330	−1.380	39.800	4.430
	7SJ3	−9.2	0.161	0.696	−61.330	−0.930	0.275	6.570
	8T35	−7.6	0.133	0.575	−50.670	−1.130	2.170	5.430
	3NAR	−6.1	0.107	0.461	−40.670	−1.400	31.300	4.360
Catechin	1D5R	−8.0	0.229	0.676	4.790	7.310	0.576	5.710
	4P7U	−7.1	0.203	0.600	4.250	8.240	6.140	5.070
	7SJ3	−9.3	0.266	0.785	5.570	6.290	0.275	6.640
	8T35	−7.4	0.211	0.625	4.430	7.900	2.170	5.290
	3NAR	−5.7	0.154	0.456	3.230	10.830	122.000	3.860
Rutin	1D5R	−8.4	0.115	0.501	−7.370	−9.910	0.576	6.000
	4P7U	−7.2	0.099	0.429	−6.320	−11.560	6.140	5.143
	7SJ3	−9.2	0.126	0.544	−8.070	−9.120	0.275	6.643
	8T35	−7.0	0.096	0.417	−6.140	−11.880	16.300	5.000
	3NAR	−7.3	0.100	0.436	−6.400	−11.400	5.420	5.214

Among the compounds, alpha‐pinene exhibited its strongest interaction with 7SJ3, showing a binding energy of −7.0 kcal/mol, a ligand efficiency (LE) of 26.9%, and a ligand lipophilic efficiency (LLE) of 1.456. The compound also achieved a low inhibition constant (*K*_i_) of 1.98 μM for 7SJ3, indicating a strong affinity, while its interactions with other proteins demonstrated moderate binding energies ranging from −5.1 to −5.9 kcal/mol.

Caryophyllene showed the highest binding affinity for 7SJ3 with a binding energy of −7.7 kcal/mol, a ligand efficiency of 19.7%, and an LLE value of 1.315. However, its interactions with 8T35 and 3NAR were weaker, as reflected in the lower binding energies of −5.7 and −6.0 kcal/mol, respectively.

Humulene demonstrated its strongest binding with 7SJ3, with a binding energy of −8.8 kcal/mol, a ligand efficiency of 22.6%, and an LLE value of 5.271, accompanied by a low *K*_i_ value of 7.403 μM. For other proteins, Humulene showed good binding energies, ranging from −6.1 to −7.7 kcal/mol, and consistently high LLE values, suggesting its strong overall binding potential.

(E)‐beta‐farnesene, on the other hand, exhibited weaker binding interactions, with its strongest affinity observed for 7SJ3 at −6.7 kcal/mol and an LLE of 1.188. The weakest interaction was with 3NAR, where it achieved a binding energy of −4.8 kcal/mol and a *K*_i_ of 393.3 μM, indicating a significantly low binding affinity.

alpha‐Cadinene showed notable binding interactions with 7SJ3, achieving a binding energy of −8.8 kcal/mol, a ligand efficiency of 22.6%, and an LLE of 5.270. It also displayed moderate binding with 1D5R and 4P7U, with binding energies of −7.1 and −6.6 kcal/mol, respectively.

Oleuropein achieved its highest binding affinity with 7SJ3, showing a binding energy of −9.2 kcal/mol, a ligand efficiency of 16.1%, and an LLE of −0.93. While Oleuropein exhibited strong binding with 7SJ3, its interactions with other proteins were moderate, with binding energies ranging from −6.1 to −8.0 kcal/mol.

Catechin exhibited the highest binding energy of −9.3 kcal/mol with 7SJ3, along with a ligand efficiency of 26.6% and a *K*_i_ of 6.29 μM. Catechin demonstrated broad binding potential, with binding energies for other proteins ranging from −5.7 to −8.0 kcal/mol.

Similarly, rutin showed its strongest interaction with 7SJ3, with a binding energy of −9.2 kcal/mol, a ligand efficiency of 12.6%, and a *K*_i_ of 0.275 μM. Interactions with other proteins, such as 3NAR (−7.3 kcal/mol), displayed moderate affinity with a *K*_i_ of 5.42 μM.

Catechin, humulene, and rutin exhibited the strongest interactions with this protein, highlighting their pharmacological potential. Oleuropein and alpha‐cadinene also showed consistent binding interactions across multiple targets. These results suggest that the tested compounds, particularly catechin, rutin, and humulene, have significant potential for therapeutic applications and warrant further investigation.

In this study, the binding interactions of humulene, oleuropein, catechin, and rutin with the target proteins 1D5R, 4P7U, 7SJ3, 8T35, and 3NAR were investigated through molecular docking simulations to understand their potential pharmacological significance (Table 7). Each compound exhibited specific binding modes, characterized by various interaction types such as conventional hydrogen bonds, carbon–hydrogen bonds, pi‐sulfur, pi–anion, amide–pi stacking, and pi–alkyl interactions. The detailed analysis provided insights into the structural basis of their binding affinities and mechanisms of action.

**TABLE 7:** Docking of predicted interactions of docked conformations of compounds against proteins.

Ligand	Protein	Amino acids	Interacting	Distance
Humulene	4P7U	A:GLN41:HN ‐ :[001:N4	Conventional hydrogen bond	2.11
		:[001:H3 ‐ A:GLN41:O	Carbon–hydrogen bond	3.04
		:[001:H10 ‐ :[001:O2	Carbon–hydrogen bond	2.25
		A:ASP39:OD1 ‐ :[001	pi–anion	4.05
		A:CYS38:SG ‐ :[001	pi–sulfur	4.29
		A:CYS44:SG ‐ :[001	pi–sulfur	5.07
		:[001:S2 ‐ A:PHE126	pi–sulfur	3.30
		A:CYS38:C, O; ASP39:N ‐ :[001	Amide–pi stacked	4.20
	3NAR	A:LYS667:HZ2 ‐ :[001:O1	Conventional hydrogen bond	1.46
		A:LYS676:HZ1 ‐ :[001:O4	Conventional hydrogen bond	1.91
		A:TRP722:HE1 ‐ :[001:S2	Conventional hydrogen bond	2.02
		A:TRP707:HD1 ‐ :[001:O1	Carbon–hydrogen bond	2.54
		:[001:H1 ‐ :[001:O1	Carbon–hydrogen bond	2.25
		:[001:H2 ‐ A:ASP710:OD2	Carbon–hydrogen bond	2.23
		:[001:H4 ‐ :[001:N4	Carbon–hydrogen bond	2.09
		:[001:S1 ‐ A:TRP707	pi–sulfur	5.47
		:[001:S2 ‐ A:TRP722	pi–sulfur	4.18
		:[001:S2 ‐ A:TRP722	pi–sulfur	5.32
		:[001 ‐ :[001	pi–pi T‐shaped	5.73
		A:ASP710:C, O; THR711:N ‐ :[001	Amide–pi stacked	5.13
		A:ALA714 ‐ :[001:CL1	Alkyl	3.56
		:[001:C1 ‐ A:CYS665	Alkyl	4.60
		:[001:C1 ‐ A:LYS666	Alkyl	4.15
		:[001:C1 ‐ A:LYS667	Alkyl	4.45
		:[001:CL1 ‐ A:LEU720	Alkyl	4.84
		:[001 ‐ A:LEU672	pi–alkyl	4.23
		:[001 ‐ A:LEU720	pi–alkyl	5.39
Oleuropein	1D5R	:[001:H2 ‐ A:ASN329:OD1	Conventional hydrogen bond	2.36
		:[001:H3 ‐ A:ASN323:O	Conventional hydrogen bond	1.52
		:[001:H8 ‐ A:TYR176:O	Conventional hydrogen bond	2.48
		:[001:H9 ‐ A:TYR176:O	Conventional hydrogen bond	2.42
		:[001:H29 ‐ A:VAL166:O	Conventional hydrogen bond	2.29
		A:PRO169:HD2 ‐ :[001:O4	Carbon–hydrogen bond	2.85
		A:ARG173:HA ‐ :[001:O10	Carbon–hydrogen bond	2.32
		:[001:H16 ‐ A:ARG173:O	Carbon–hydrogen bond	2.74
		:[001:H22 ‐ A:TYR176:OH	Carbon–hydrogen bond	2.96
		:[001:H24 ‐ :[001:O1	Carbon–hydrogen bond	2.14
		:[001:H25 ‐ A:THR167:O	Carbon–hydrogen bond	2.40
		:[001:H26 ‐ :[001:O6	Carbon–hydrogen bond	2.42
		:[001:H27 ‐ A:ASN329:OD1	Carbon–hydrogen bond	2.51
		:[001:H32 ‐ A:THR167:O	Carbon–hydrogen bond	2.61
		:[001:C12 ‐ A:PRO169	Alkyl	4.47
	7SJ3	A:TYR17:HH ‐ :[001:O5	Conventional hydrogen bond	2.04
		A:LYS35:HZ2 ‐ :[001:O10	Conventional hydrogen bond	1.98
		:[001:H2 ‐ A:ASP158:OD2	Conventional hydrogen bond	1.69
		:[001:H3 ‐ A:ASN145:OD1	Conventional hydrogen bond	2.37
		:[001:H3 ‐ A:ASP158:OD2	Conventional hydrogen bond	1.67
		:[001:H8 ‐ A:GLU94:O	Conventional hydrogen bond	1.63
		:[001:H9 ‐ A:GLU94:O	Conventional hydrogen bond	2.53
		:[001:H29 ‐ A:VAL14:O	Conventional hydrogen bond	2.07
		A:ASP158:HA ‐ :[001:O10	Carbon–hydrogen bond	2.48
		:[001:H21 ‐ A:GLU144:O	Carbon–hydrogen bond	2.52
		:[001:H24 ‐ :[001:O4	Carbon–hydrogen bond	1.78
		:[001:H14 ‐ A:PHE93	pi–sigma	2.14
		A:PHE93 ‐ :[001	pi–pi stacked	4.69
		A:ALA157 ‐ :[001:C24	Alkyl	2.96
		:[001:C24 ‐ A:LEU147	Alkyl	3.56
		A:TYR17 ‐ :[001:C12	pi–alkyl	3.41
		:[001 ‐ A:ALA33	pi–alkyl	3.80
		:[001 ‐ A:VAL72	pi–alkyl	4.74
		:[001 ‐ A:LEU147	pi–alkyl	5.41

	3NAR	A:GLN683:HE21 ‐ :[001:O5	Conventional hydrogen bond	2.28
		A:GLN683:HE22 ‐ :[001:O1	Conventional hydrogen bond	2.17
		A:GLN683:HE22 ‐ :[001:O13	Conventional hydrogen bond	2.09
		A:GLN727:HE21 ‐ :[001:O7	Conventional hydrogen bond	1.66
		:[001:H1 ‐ B:ASN730:O	Conventional hydrogen bond	2.84
		:[001:H3 ‐ A:ARG681:O	Conventional hydrogen bond	1.67
		:[001:H9 ‐ A:VAL680:O	Conventional hydrogen bond	1.64
		:[001:H29 ‐ B:GLN727:O	Conventional hydrogen bond	2.37
		B:SER731:HA ‐ :[001:O2	Carbon–hydrogen bond	2.29
		:[001:H15 ‐ :[001:O12	Carbon–hydrogen bond	2.59
		:[001:H22 ‐ B:ARG681:O	Carbon–hydrogen bond	2.74
		:[001:H26 ‐ A:ARG681:O	Carbon–hydrogen bond	2.72
		:[001:H30 ‐ B:GLN727:O	Carbon–hydrogen bond	2.54
A:TYR723 ‐ :[001:C12		pi–alkyl	5.07
Catechin	1D5R	A:ARG173:HH22 ‐ :[001:O6	Conventional hydrogen bond	1.89
		A:TYR177:HA ‐ :[001:O1	Carbon–hydrogen bond	2.66
		A:ASP324:OD1 ‐ :[001	pi–anion	4.24
		A:TYR177 ‐ :[001	pi–pi T‐shaped	4.57
		A:ARG173 ‐ :[001	Alkyl	3.76
		A:TYR177 ‐ :[001	pi–alkyl	5.48
		:[001 ‐ A:ARG173	pi–alkyl	4.74
		:[001 ‐ A:ARG172	pi–alkyl	4.83
		:[001 ‐ A:ARG173	pi–alkyl	4.82
	7SJ3	A:HIS95:HD1 ‐ :[001:O1	Conventional hydrogen bond	2.35
		A:ASP158:HN ‐ :[001:O6	Conventional hydrogen bond	2.04
		:[001:H2 ‐ A:VAL96:O	Conventional hydrogen bond	2.10
		:[001:H4 ‐ A:GLU94:O	Conventional hydrogen bond	1.91
		:[001:H8 ‐ A:ASN145:OD1	Conventional hydrogen bond	1.57
		:[001:H8 ‐ A:ASP158:OD2	Conventional hydrogen bond	3.02
		A:HIS95:HA ‐ :[001:O1	Carbon–hydrogen bond	2.92
		A:ASP158:OD1 ‐ :[001	pi–anion	4.63
		A:ALA33 ‐ :[001	Alkyl	4.96
		A:VAL72 ‐ :[001	Alkyl	4.50
		A:LEU147‐:[001	Alkyl	5.00
		A:ALA157 ‐ :[001	Alkyl	3.85
		A:PHE93 ‐ :[001	pi–alkyl	4.74
		:[001 ‐ A:ALA33	pi–alkyl	3.66
		:[001 ‐ A:VAL72	pi–alkyl	5.23


		:[001 ‐ A:VAL96	pi–alkyl	5.12
		:[001 ‐ A:LEU147	pi–alkyl	4.97
		:[001 ‐ A:ALA157	pi–alkyl	5.44
		:[001 ‐ A:VAL20	pi–alkyl	4.61
	:[001 ‐ A:ALA157	pi–alkyl	4.93
	8T35	A:ARG10:HH12 ‐ :[001:O6	Conventional hydrogen bond	2.30
		A:ARG37:HH11 ‐ :[001:O1	Conventional hydrogen bond	2.42
		:[001:H2 ‐ A:TYR38:O	Conventional hydrogen bond	2.93
		:[001:H2 ‐ A:LYS39:O	Conventional hydrogen bond	1.59
		:[001:H4 ‐ A:ASP4:O	Conventional hydrogen bond	2.07
		:[001:H7 ‐ A:LEU47:O	Conventional hydrogen bond	1.48
		:[001:H8 ‐ A:ILE35:O	Conventional hydrogen bond	2.25
		:[001:H11 ‐ A:GLU36:OE2	Conventional hydrogen bond	2.10
		A:ARG37 ‐ :[001	Alkyl	5.07
:[001 ‐ A:ARG37		pi–alkyl	4.71
:[001 ‐ A:VAL46		pi–alkyl	4.59
Rutin	1D5R	A:GLN149:HE22 ‐ :[001:O7	Conventional hydrogen bond	2.64
		A:ARG172:HE ‐ :[001:O4	Conventional hydrogen bond	2.24
		:[001:H12 ‐ A:TYR176:OH	Conventional hydrogen bond	1.63
		:[001:H19 ‐ A:ASN329:OD1	Conventional hydrogen bond	2.11
		:[001:H20 ‐ A:ASN323:O	Conventional hydrogen bond	2.37
		:[001:H21 ‐ A:ASP324:O	Conventional hydrogen bond	2.00
		:[001:H26 ‐ A:ASP324:OD1	Conventional hydrogen bond	2.20
		A:ARG172:HD2 ‐ :[001:O4	Carbon–hydrogen bond	2.59
		A:ARG172:HD2 ‐ :[001:O5	Carbon–hydrogen bond	1.78
		:[001:H1 ‐ :[001:O13	Carbon–hydrogen bond	2.55
		:[001:H3 ‐ :[001:O13	Carbon–hydrogen bond	1.69
		:[001:H7 ‐ A:PRO169:O	Carbon–hydrogen bond	2.79
		:[001:H8 ‐ A:ASP324:OD1	Carbon–hydrogen bond	2.55
		:[001:H9 ‐ A:PRO169:O	Carbon–hydrogen bond	2.48
		A:TYR177 ‐ :[001:C1	pi–alkyl	5.11
	4P7U	:[001:H12 ‐ A:GLN41:O	Conventional hydrogen bond	1.81
		:[001:H13 ‐ A:GLN41:O	Conventional hydrogen bond	1.44
		:[001:H19 ‐ A:THR109:OG1	Conventional hydrogen bond	2.48
		:[001:H21 ‐ A:PHE126:O	Conventional hydrogen bond	1.78
		:[001:H25 ‐ A:ASP39:OD1	Conventional hydrogen bond	2.90
		A:ASN40:HA ‐ :[001:O7	Carbon–hydrogen bond	2.85
		A:ASP39:OD1 ‐ :[001	pi–anion	3.85


		:[001:H20 ‐ A:PHE111	pi–donor hydrogen bond	2.86
		A:CYS38:SG ‐ :[001	pi–sulfur	5.30
		A:PHE126 ‐ :[001	pi–pi stacked	4.20
		A:TRP65 ‐ :[001	pi–pi T‐shaped	4.55
	:[001 ‐ A:LYS67	pi–alkyl	4.82
	7SJ3	:[001:H17 ‐ A:GLU94:O	Conventional hydrogen bond	1.70
		:[001:H19 ‐ A:VAL96:O	Conventional hydrogen bond	2.48
		:[001:H21 ‐ A:ILE12:O	Conventional hydrogen bond	2.48
		:[001:H25 ‐ A:ASP97:O	Conventional hydrogen bond	2.15
		:[001:H27 ‐ A:VAL96:O	Conventional hydrogen bond	2.24
		A:GLN98:HA ‐ :[001:O1	Carbon–hydrogen bond	2.56
		:[001:H1 ‐ :[001:O3	Carbon–hydrogen bond	2.55
		:[001:H1 ‐ :[001:O5	Carbon–hydrogen bond	3.05
		:[001:H2 ‐ A:VAL96:O	Carbon–hydrogen bond	2.92
		:[001:H6 ‐ A:GLU144:O	Carbon–hydrogen bond	2.43
		:[001:H7 ‐ A:ILE12:O	Carbon–hydrogen bond	2.61
		:[001:H9 ‐ A:ILE12:O	Carbon–hydrogen bond	2.14
		:[001:H23 ‐ A:ASP99:OD2	Carbon–hydrogen bond	2.13
		A:PHE93 ‐ :[001	pi–pi stacked	5.51
		:[001:C1 ‐ A:VAL96	Alkyl	4.23
		:[001:C1 ‐ A:LEU147	Alkyl	3.55
		:[001 ‐ A:VAL20	pi–alkyl	4.89
		:[001 ‐ A:ALA33	pi–alkyl	4.58
		:[001 ‐ A:LEU147	pi–alkyl	4.87
		:[001 ‐ A:ALA157	pi–alkyl	4.76
		:[001 ‐ A:ALA33	pi–alkyl	3.92
		:[001 ‐ A:VAL72	pi–alkyl	4.82
		:[001 ‐ A:VAL96	pi–alkyl	4.93
		:[001 ‐ A:LEU147	pi–alkyl	4.89
		:[001 ‐ A:ALA157	pi–alkyl	5.27
:[001 ‐ A:VAL20		pi–alkyl	4.71
:[001 ‐ A:ALA157		pi–alkyl	4.80

	8T35	A:GLN116:HE21 ‐ :[001:O14	Conventional hydrogen bond	2.28
		B:PHE79:HN ‐ :[001:O5	Conventional hydrogen bond	2.03
		:[001:H13 ‐ B:SER61:O	Conventional hydrogen bond	2.04
		:[001:H17 ‐ B:PRO75:O	Conventional hydrogen bond	2.25
		:[001:H20 ‐ B:PHE79:O	Conventional hydrogen bond	1.65
		:[001:H25 ‐ A:GLU117:OE1	Conventional hydrogen bond	1.87
		:[001:H26 ‐ A:GLU117:OE1	Conventional hydrogen bond	2.33
		B:ALA78:HA ‐ :[001:O5	Carbon–hydrogen bond	3.02
		:[001:H1 ‐ :[001:O3	Carbon–hydrogen bond	2.64
		:[001:H4 ‐ A:GLU117:OE1	Carbon–hydrogen bond	2.29
		:[001:H5 ‐ A:GLN116:O	Carbon–hydrogen bond	2.11
		:[001:H6 ‐ :[001:O1	Carbon–hydrogen bond	2.34
		:[001:H10 ‐ B:PHE79:O	Carbon–hydrogen bond	2.31
		:[001:H10 ‐ :[001:O5	Carbon–hydrogen bond	2.47
		B:ARG68:NH1 ‐ :[001	pi–cation	3.07
		B:ARG68:NH1 ‐ :[001	pi–cation; pi–donor hydrogen bond	3.39
		:[001 ‐ B:ALA78	pi–alkyl	4.86
		:[001 ‐ B:VAL64	pi–alkyl	4.25
		:[001 ‐ B:LYS65	pi–alkyl	4.63
	3NAR	A:GLN683:HE21 ‐ :[001:O5	Conventional hydrogen bond	2.60
		A:GLN683:HE21 ‐ :[001:O15	Conventional hydrogen bond	2.80
		A:GLN727:HE21 ‐ :[001:O4	Conventional hydrogen bond	2.28
		:[001:H17 ‐ B:ASN730:OD1	Conventional hydrogen bond	2.76
		:[001:H21 ‐ A:ASN730:OD1	Conventional hydrogen bond	2.12
		:[001:H22 ‐ A:ASN730:OD1	Conventional hydrogen bond	1.52
		:[001:H25 ‐ A:ARG681:O	Conventional hydrogen bond	1.64
		:[001:H26 ‐ A:VAL680:O	Conventional hydrogen bond	2.12
		A:GLN727:HA ‐ :[001:O12	Carbon hydrogen bond	2.75
		A:SER731:HB2 ‐ :[001:O8	Carbon hydrogen bond	2.67
		:[001:H3 ‐ :[001:O3	Carbon hydrogen bond	2.71
		B:GLN683:HN ‐ :[001	pi–donor hydrogen bond	3.09
B:GLN727:HE21 ‐ :[001		pi–donor hydrogen bond	2.92

Humulene showed strong binding interactions with 4P7U, forming conventional hydrogen bonds, such as GLN41:HN and N4, with a distance of 2.11 Å, and carbon–hydrogen bonds with GLN41:O at 3.04 Å. Additional interactions included pi–sulfur with CYS38:SG (4.29 Å) and amide–pi stacking with CYS38:C, O and ASP39:N (4.20 Å), indicating a stable binding conformation. In 3NAR, humulene formed conventional hydrogen bonds with LYS667:HZ2 (1.46 Å), LYS676:HZ1 (1.91 Å), and TRP722:HE1 (2.02 Å), alongside multiple carbon–hydrogen and pi–sulfur interactions, further supporting its binding stability. These interactions highlight the compound's potential for targeting proteins involved in critical biological pathways. Figures 10A and 13A show the interactions of humulene with type II transforming growth factor beta receptor (4P7U) and ZHX1 HD4 (zinc‐fingers and homeoboxes protein 1, homeodomain 4) (3NAR), respectively.

**FIGURE 9:**
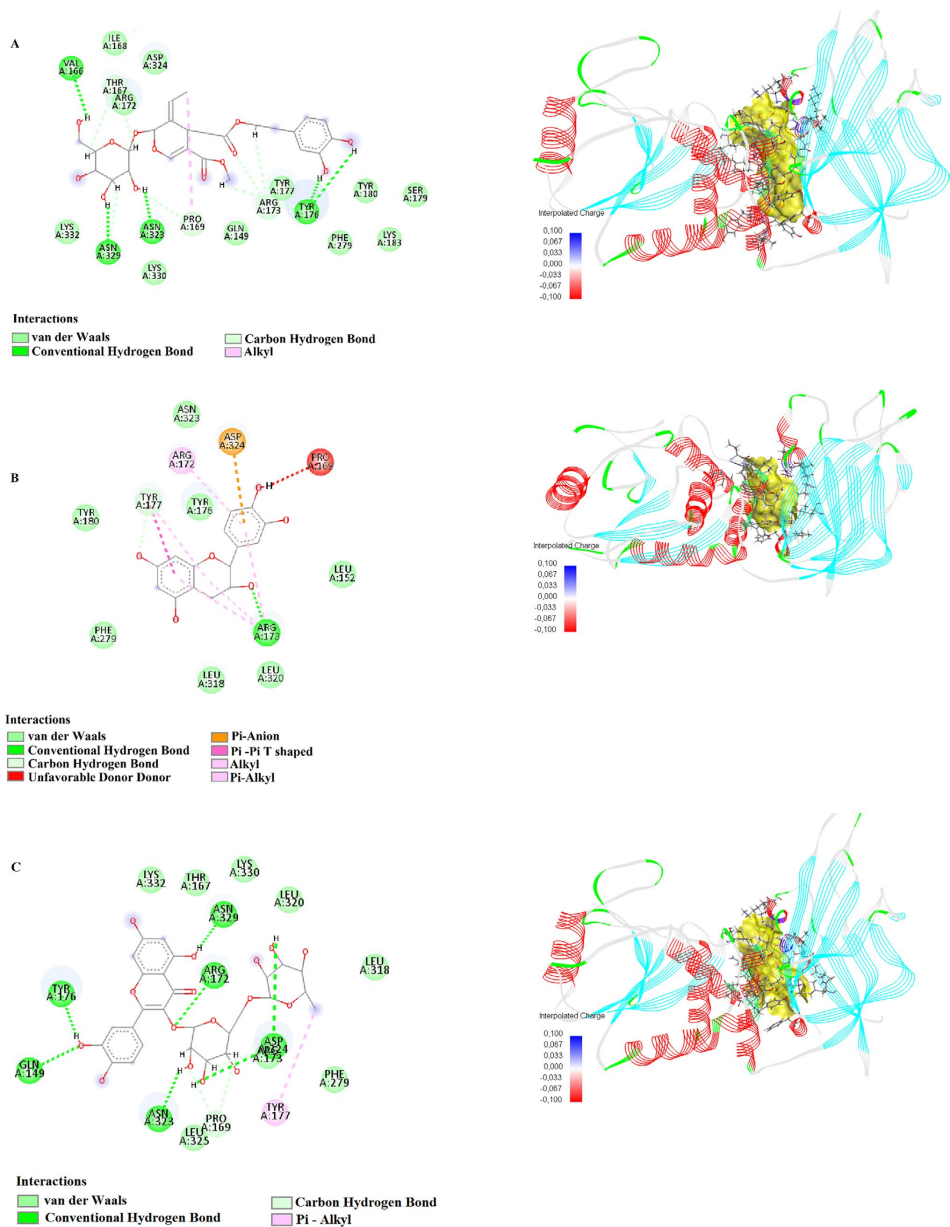
2D and 3D interactions of (A) oleuropein, (B) catechin, and (C) rutin with PTEN tumor suppressor (1D5R).

Oleuropein displayed robust binding affinity to 1D5R, forming conventional hydrogen bonds with ASN329:OD1 (2.36 Å) and TYR176:O (2.42 Å), and carbon‐hydrogen bonds with PRO169:HD2 (2.85 Å). Additional interactions included alkyl contacts with PRO169:C12 (4.47 Å), contributing to the compound's stable binding. In 7SJ3, Oleuropein formed tight hydrogen bonds with ASP158:OD2 (1.67 Å) and GLU94:O (1.63 Å), and pi–sigma interactions with PHE93 (2.14 Å), underscoring its adaptability to bind diverse protein sites. The compound also formed significant amide–pi stacking interactions, enhancing its binding efficacy. Figures 9A, 11A, and 13B show the interactions of oleuropein with PTEN tumor suppressor (1D5R), CDK4‐cyclin D3 bound to abemaciclib (7SJ3), and ZHX1 HD4 (zinc‐fingers and homeoboxes protein 1, homeodomain 4) (3NAR), respectively.

Catechin demonstrated strong interactions with 1D5R, where it formed hydrogen bonds with ARG173:HH22 (1.89 Å) and TYR177:HA (2.66 Å), along with pi–alkyl interactions with TYR177:C1 (5.48 Å). In 7SJ3, catechin exhibited hydrogen bonds with ASP158:HN (2.04 Å) and VAL96:O (2.10 Å), as well as pi‐anion interactions with ASP158:OD1 (4.63 Å), suggesting a highly stable binding configuration. Notably, its interactions spanned diverse amino acid residues, highlighting its broad binding potential. Figures 9B, 11B, and 12A show the interactions of catechin with PTEN tumor suppressor (1D5R), CDK4‐cyclin D3 bound to abemaciclib (7SJ3), and K51 acetylated LC3A in complex with the LIR of TP53INP2/DOR (8T35), respectively.

**FIGURE 10:**
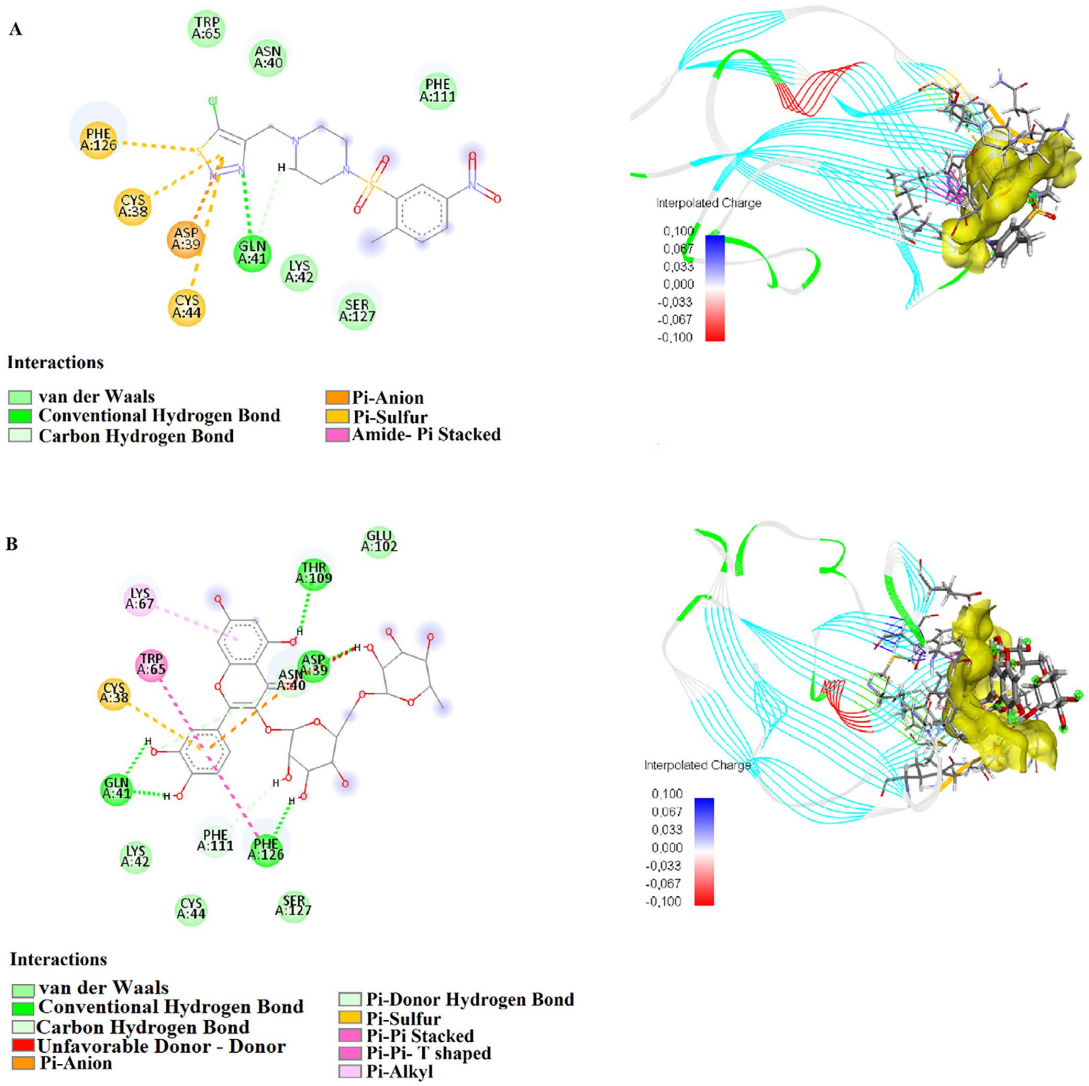
2D and 3D interactions of (A) humulene and (B) rutin with type II transforming growth factor beta receptor (4P7U).

**FIGURE 11:**
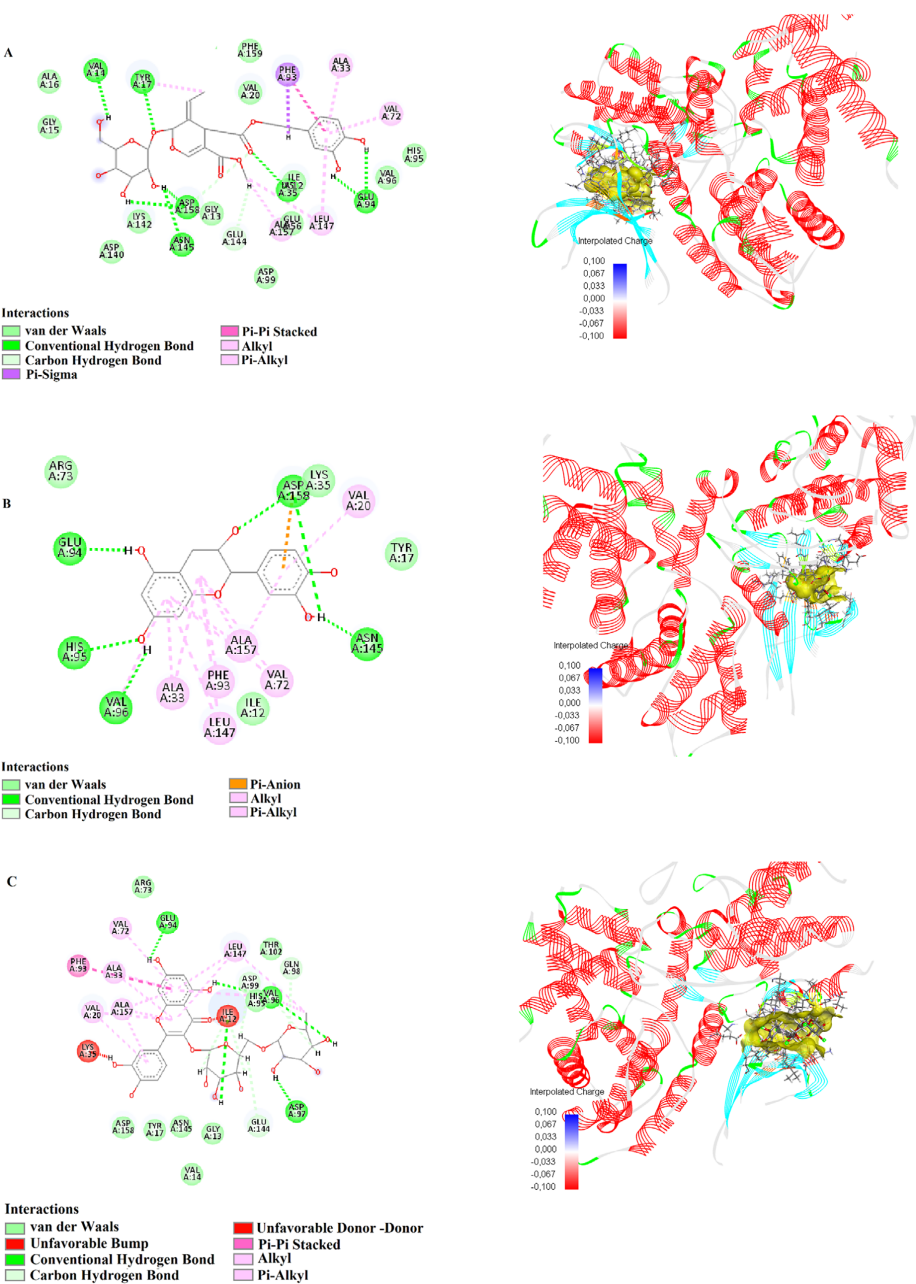
2D and 3D interactions of (A) oleuropein, (B) catechin, and (C) rutin with CDK4‐cyclin D3 bound to abemaciclib (7SJ3).

**FIGURE 12:**
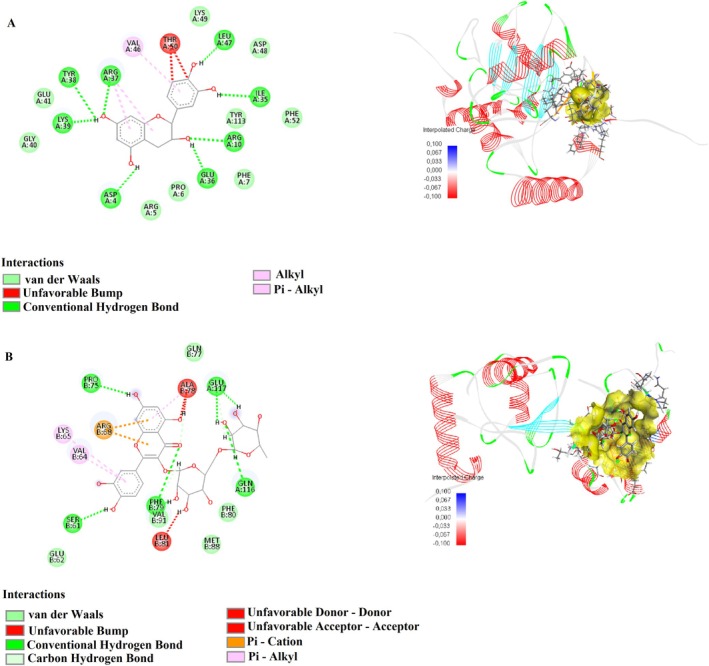
2D and 3D interactions of (A) catechin and (B) rutin with K51 acetylated LC3A in complex with the LIR of TP53INP2/DOR (8T35).

**FIGURE 13:**
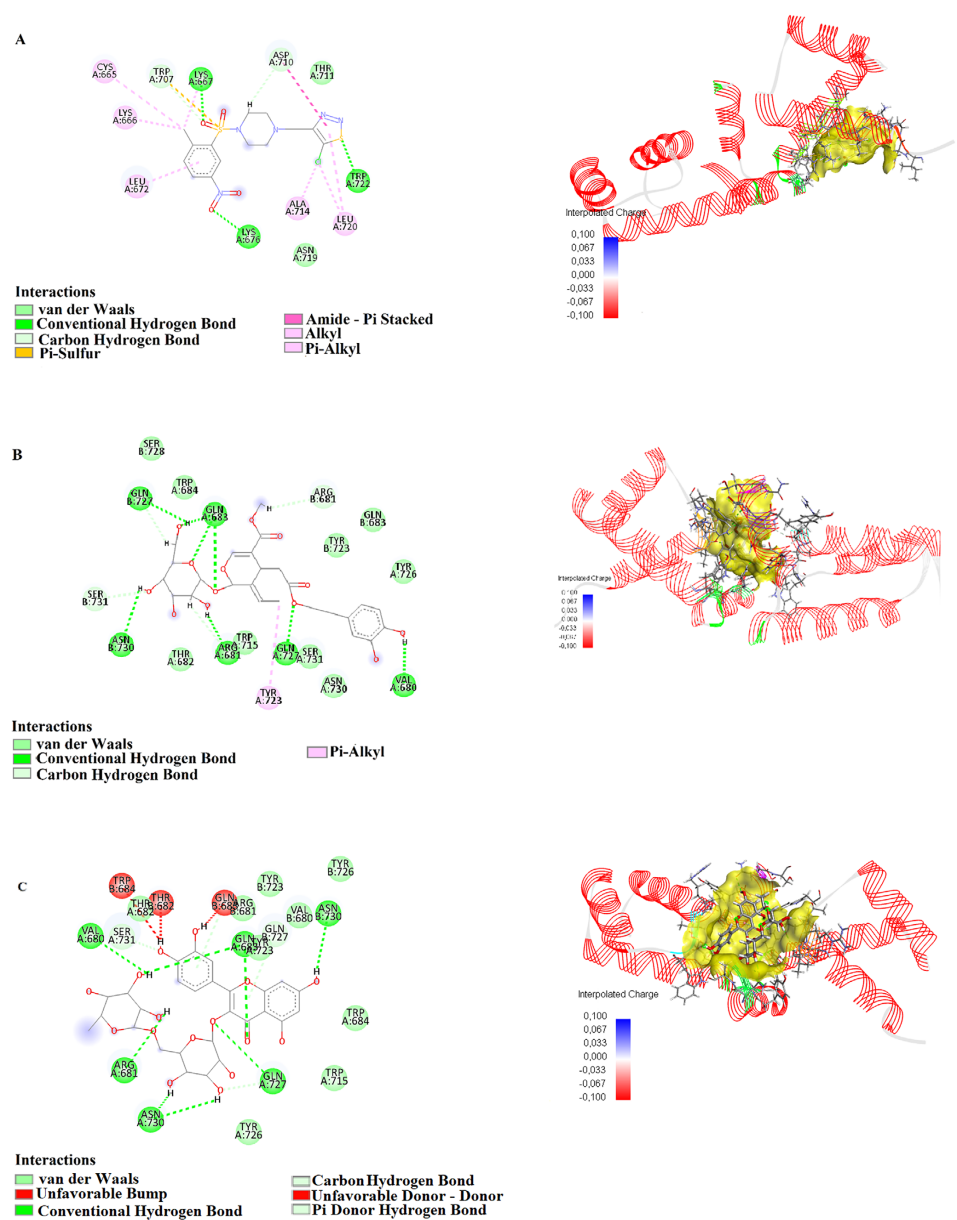
2D and 3D interactions of (A) humulene, (B) oleuropein, and (C) rutin with ZHX1 HD4 (zinc‐fingers and homeoboxes protein 1, homeodomain 4) (3NAR).

Rutin showed the most complex interaction network, engaging with multiple amino acids in both 1D5R and 7SJ3. In 1D5R, it formed hydrogen bonds with GLN149:HE22 (2.64 Å), ARG172:HE (2.24 Å), and TYR176:OH (1.63 Å), complemented by pi–alkyl interactions with TYR177:C1 (5.11 Å). In 7SJ3, Rutin exhibited hydrogen bonds with GLU94:O (1.70 Å), VAL96:O (2.48 Å), and ASP97:O (2.15 Å), alongside pi–pi stacked interactions with PHE93 (5.51 Å). Its binding distances, ranging from 1.44 to 5.51 Å, indicate strong and specific interactions, supported by its highly polar nature. Figures 9, 10B, 11C, 12B, and 13C show the interactions of rutin with PTEN tumor suppressor (1D5R), type II transforming growth factor beta receptor (4P7U), CDK4‐cyclin D3 bound to abemaciclib (7SJ3), K51 acetylated LC3A in complex with the LIR of TP53INP2/DOR (8T35), and ZHX1 HD4 (zinc‐fingers and homeoboxes protein 1, homeodomain 4) (3NAR), respectively.

The diversity of interactions observed across the compounds, including hydrogen bonds, pi–sulfur, pi–anion, and pi–alkyl contacts, underscores their potential for targeting various protein sites. Binding distances, generally ranging between 1.5 and 5.5 Å, suggest strong and stable interactions, while the range of binding affinities reflects the structural and functional diversity of these compounds.

These findings provide a detailed molecular basis for the potential therapeutic applications of humulene, oleuropein, catechin, and rutin, highlighting their versatility and significance in drug discovery research.

The cytotoxic effect of *Stachys pilifera* extract on HepG2 cells increased over time, as evidenced by a decrease in IC_50_ values from 128.49 μg/mL at 24 h to 107.11 μg/mL at 72 h (Barmoudeh et al. 2022). Similarly, the IC_50_ value of *B. macrantha* ( *Stachys macrantha* ) extract on MDA‐MB‐231 cells was determined to be 0.8 mg/mL after 72 h, and it was found to inhibit cell viability in MDA cells in a dose‐dependent manner. Based on the reduction in IC_50_ values, *S. pilifera* extract may exhibit a strong cytotoxic effect. In a study by Kokhdan et al. (2018), the methanol extract of *S. pilifera* demonstrated positive inhibitory effects on cell viability and significant antiproliferative activity against the HT‐29 cell line (colon adenocarcinoma). Furthermore, the chloroform extract of *Stachys laxa* significantly inhibited the proliferation of both HT‐29 and T47D (ductal carcinoma) cell lines, and the total extract of *Stachys* *subaphylla* also exhibited antiproliferative properties against the T47D cell line (Khanavi et al. 2012). In addition, Háznagy‐Radnai et al. (2008) observed that the methanolic extracts of certain *Stachys* species, such as *Stachys palustris* and *Stachys recta* , showed significant antiproliferative activity against cervix adenocarcinoma (HeLa) cells, while the flowers of *S. germanica* exhibited effectiveness against breast adenocarcinoma (MCF‐7) cells. Moreover, Lachowicz et al. (2020) reported that the methanol extracts of *Sanguisorba officinalis* L. leaves and flowers demonstrated significant antiproliferative effects against bladder cancer (HCV29T), colorectal adenocarcinoma (DLD‐1), pancreatic ductal adenocarcinoma (BxPC3), and Jurkat cell lines. The leaf and flower extracts of *S. palustris* significantly reduced cell viability in human lung, pancreatic, bladder, and colon cancer cell lines, as well as in human acute myeloid leukemia cells (Lachowicz‐Wiśniewska et al. 2022). In our study, the results of the MTT assay indicated that treatment with different concentrations of *B. macrantha* for 72 h inhibited the proliferation of MDA cells. The dichloromethane extract of *S. circinata* exhibited anti‐proliferative properties, inhibiting cell growth in a dose‐dependent manner in MCF7 and HepG2 cells. The extract showed selective cytotoxicity against cancer cell lines, with IC_50_ values of 3.67 and 4.87 mg/mL for MCF7 and HepG2 cells, respectively (Slimani et al. 2023). Similarly, *B. macrantha* extract inhibited cell viability in MDA cells in a dose‐dependent manner, with an IC_50_ value of 0.8 mg/mL in MDA‐MB‐231 cells. These results demonstrate the cytotoxic effects of both extracts on cancer cells and their ability to inhibit cell growth. *Stachys spreitzenhoferi* extract showed antiproliferative activity against acute myeloid leukemia (U937) cells, with an IC_50_ value of 0,75 mg/mL (Napolitano et al. 2022). On the other hand, *B. macrantha* extract exhibited an IC_50_ value of 0.8 mg/mL on MDA‐MB‐231 cells. While both extracts demonstrated antiproliferative effects on cancer cell lines, the IC_50_ values differ depending on the cell type. These findings suggest that the effects of both plant extracts may vary across different cancer types. The hydroalcoholic extract of *Stachys setifera* showed an IC_50_ value of 827.52 μg/mL after 24 h in MCF‐7 cells (Panahi Kokhdan et al. 2021). In contrast, the extract of *B. macrantha* exhibited an IC_50_ value of 0.8 mg/mL after a 2‐h treatment in MDA‐MB‐231 cells. These results demonstrate that both plant extracts exhibit antiproliferative effects on cancer cells, but their efficacy varies depending on the cell type and treatment duration. The hydroalcoholic extract of *S. setifera* showed a higher cytotoxic effect in MCF‐7 cells, while the extract of *B. macrantha* exhibited a lower cytotoxic effect in MDA‐MB‐231 cells. These findings suggest that the effects of both extracts on cancer cells may be cell type‐ and time‐dependent.

microRNAs (miRNAs) are small, non‐coding RNA sequences that play a crucial role in the post‐transcriptional regulation of gene expression, primarily by promoting mRNA degradation or repressing translation. Depending on the effects they produce, the genes they target, and the tissue in which they operate, miRNAs can function as either tumor suppressors or oncogenic drivers (Croce 2009). Research on miRNAs emphasizes their involvement in disease mechanisms, their potential as diagnostic markers, and their use in therapeutic strategies, particularly in advancing our understanding of cancer, neurological, cardiovascular, and autoimmune disorders (Condrat et al. 2020).

After the application of the plant extract to cancer cells, significant increases in the expression of miR‐19b and miR‐20a were observed in MDA cells. This unexpected response requires further interpretation, because miR‐19b and miR‐20b functions as oncomirs have been shown to promote cancer proliferation and migration (Cai et al. 2015; Gu et al. 2017). The extract may have differential effects on various miRNAs. The increase in miR‐19b and miR‐20a might be counterbalanced by the regulatory effects on other tumor‐suppressive miRNAs, such as miR‐126 and miR‐155. While the upregulation of miR‐19b and miR‐20a may indicate an undesired proliferative effect on cancer cells, the overall impact of the extract appears to be more complex. We need a detailed analysis of target gene expression and modulation of miR‐19b and miR‐20a‐specific targets.

miR‐126‐3p, which is commonly downregulated in breast cancers, showed increased levels in sorafenib‐treated breast cancer cell Pelisenco et al. (2024), consistent with our findings of the extract‐treated group of MDA cells. This upregulation may lead to the inhibition of cancer cell proliferation, migration, and invasion. In our study, a decrease in miR‐155 expression was observed in MDA‐MB‐231 cells following extract treatment. This finding aligns with the results of Mizielska et al. 2023, who reported reduced miR‐155 expression in MCF7 cells after doxorubicin treatment. However, Mizielska et al. (2023) also noted an increase in miR‐155 expression in MDA‐MB‐231 cells following treatment with doxorubicin and cisplatin. This observation contrasts with the decreasing trend seen in our study. These contradictory results could be attributed to cell‐type‐specific mechanisms, differences in the molecular pathways affected by the treatments, or dose‐dependent effects of the compounds.

miR‐200c is generally considered a tumor‐suppressive microRNA. This microRNA inhibits epithelial–mesenchymal transition, thereby reducing the metastatic potential of cancer cells (Mutlu et al. 2016). Therefore, the observed increase in miR‐200c expression following extract treatment in MDA‐MB‐231 cells is a logical and expected outcome, aligning with its known tumor‐suppressive role.

These findings suggest that plant extracts can regulate miRNA expression, potentially restoring the balance between oncogenic and tumor‐suppressive miRNAs in cancer cells. By suppressing oncogenic miRNAs and increasing tumor‐suppressive miRNAs, these extracts may inhibit cancer cell proliferation, induce apoptosis, and reduce metastatic potential. In conclusion, such plant extracts show promise as therapeutic agents for breast cancer by positively modulating miRNA expression profiles. However, further research is necessary to fully understand their mechanisms, efficacy, and safety before clinical application (Asgharzade et al. 2020).

Despite advancements in breast cancer therapy, this disease remains one of the leading causes of female mortality worldwide. Dysregulation of miRNA plays a crucial role in the initiation and progression of cancer. In this context, the use of herbal compounds exhibiting anticancer properties through the modulation of microRNA expression presents a promising strategy for cancer treatment.

alpha‐Pinene has been found to exhibit antitumoral activity (Acikgul et al. 2024). Alpha‐pinene is an organic compound with anticancer properties and is considered a potential therapeutic agent in breast cancer (BC) treatment. miR‐21 promotes cancer cell invasion and the development of an aggressive phenotype by inhibiting PTEN. Studies have shown that miR‐21 expression significantly decreases in cells treated with alpha‐pinene compared to the control group. In contrast, PTEN gene expression exhibits an opposite pattern, showing a significant increase following alpha‐pinene treatment. These findings suggest that alpha‐pinene suppresses miR‐21 expression while enhancing PTEN expression, thereby reducing the invasion and proliferation abilities of BC cells. These properties highlight alpha‐pinene's potential as an effective therapeutic approach in breast cancer treatment (Yazdi et al. 2024).

Beta‐caryophyllene, the main component of chili pepper, is used for the prevention of various cancers. Beta‐caryophyllene from chili pepper exhibits inhibitory activity in non‐small cell lung cancer cells by targeting the sphingosine kinase 1 pathway through miR‐659‐3p (Lei et al. 2021). Beta‐caryophyllene shows cytotoxic effects on MDA‐MB‐468 cells at a lower concentration. Doxorubicin, on the other hand, is a compound with strong anticancer effects. The IC_50_ value of beta‐caryophyllene is 18.6 μg/mL, while doxorubicin has a stronger effect with an IC_50_ value of 4.7 μg/mL. Therefore, beta‐caryophyllene is approximately three times less effective than doxorubicin (Di Sotto et al. 2022).

In human colorectal cancer HT29 cells, a 24‐h treatment with γ‐humulene resulted in a dose‐dependent significant reduction in cell viability, with an IC_50_ value of 53.67 ± 2.99 μM (Lan et al. 2011). It has been reported that α‐humulene and trans‐caryophyllene extracted from *S. officinalis* essential oil inhibit tumor cell growth (El Hadri et al. 2010). alpha‐pinene and gamma‐cadinene showed cytotoxic activity against MCF‐7 (Nguyen et al. 2022).

It has been observed that oleuropein significantly reduces cell viability in a dose‐ and time‐dependent manner, while also increasing apoptosis in MCF7 and MDA‐MB‐231 cells. In the presence of oleuropein, the expression levels of miR‐125b, miR‐16, miR‐34a, p53, p21, and TNFRS10B were increased, while those of bcl‐2, mcl1, miR‐221, miR‐29a, and miR‐21 were decreased. The findings indicate that oleuropein may induce apoptosis not only by increasing the expression of pro‐apoptotic genes and tumor‐suppressor miRNAs but also by reducing the expression of anti‐apoptotic genes and oncomiRs. Consequently, oleuropein can be considered a potential herbal agent for cancer therapy (Asgharzade et al. 2020). Oleuropein has shown significant anticancer effects against gastric adenocarcinoma cells (Türkdoğan et al. 2023). Catechins exhibit antioxidant properties and may potentially demonstrate anticancer effects. The analysis showed that epigallocatechin, epigallocatechin gallate, epicatechin, and epicatechin gallate decreased the enzymatic activity of PTP1B phosphatase and reduced the viability of MCF‐7 breast cancer cells. Furthermore, epigallocatechin was identified as the most potent inhibitor of PTP1B activity (Kuban‐Jankowska et al. 2020). This study first demonstrates that epicatechin can reverse the levels of miRNAs and induce a cytostatic effect at a low concentration in MCF‐7 and HT‐29 cells (Kiran et al. 2023).

Rutin affects the expression of the microRNA known as miR‐129‐1‐3p, inhibiting growth and proliferation in mouse breast cancer cells (Li et al. 2021). Rutin significantly inhibits the proliferation of pancreatic cancer cells and induces apoptosis in these cells. This effect is associated with the upregulation of miR‐877‐3p expression, which represses the transcription of the Bcl‐2 gene. Consequently, the findings suggest that rutin plays a crucial role in combating pancreatic cancer through the rutin‐miR‐877‐3p‐Bcl‐2 axis and may serve as a potential therapeutic strategy for pancreatic cancer (Huo et al. 2022).

PTEN is a phosphatase enzyme capable of acting on both polypeptide and phosphoinositide substrates. Bioactive compounds such as oleuropein, catechin, and rutin are thought to be associated with the tumor‐suppressive effects of PTENP. Oleuropein has been reported to inhibit the PI3K/Akt signaling pathway (Han et al. 2023), thereby suppressing cell proliferation, while catechin and rutin are known to reduce oxidative stress and promote apoptosis (Bernatoniene and Kopustinskiene 2018; Wang et al. 2022), supporting this mechanism. PTENP regulates the PI3K/Akt signaling pathway by suppressing miR‐19b and plays a tumor‐suppressive role. In this context, it is suggested that compounds like oleuropein, catechin, and rutin may enhance PTENP expression or suppress the effects of miR‐19b, thereby contributing to the modulation of the PI3K/Akt pathway through PTENP, presenting a potential target for breast cancer treatment (Shi et al. 2018).

It has been found that TGFBR2 is downregulated by miR‐20a. This suggests that miR‐20a may affect the TGF‐β signaling pathway and inhibit the mesenchymal to epithelial transition process in cancer cells (De et al. 2017). Humulene is known for its anti‐inflammatory and anticancer properties (Hata Viveiros et al. 2022). Rutin, a flavonoid compound, has antioxidant and anticancer Satari et al. (2021) and may enhance the potential of miR‐20a to modulate the TGF‐β pathway. In this context, compounds such as humulene and rutin may be considered potential therapeutic agents in cancer treatment by regulating the miR‐20a‐mediated TGF‐β signaling pathway.

miR‐126 has been identified as a microRNA with significant antiproliferative effects on breast cancer cell lines. The combination of miR‐126 transfection and the CDK4/6 inhibitor ribociclib has demonstrated a stronger antitumor effect in breast cancer cell lines compared to the application of each agent alone (Andrikopoulou et al. 2021). Additionally, phytochemical compounds such as oleuropein, catechins, and rutin, which may act as CDK4‐cyclin inhibitors, are thought to exhibit strong antitumor effects in breast cancer cell lines.

miR‐155 expression is significantly higher in MCF‐7 cells compared to MDA‐MB‐231 cells. Overexpression of miR‐155 increases cell proliferation while suppressing cell apoptosis, whereas the abrogation of miR‐155 expression suppresses cell proliferation and promotes cell apoptosis in MCF‐7 cells (Zhang et al. 2013). Additionally, catechin and rutin may inhibit miR‐155 overexpression and suppress proliferation in breast cancer cells, potentially offering an alternative treatment approach.

The abnormal expression of ZEB1 has been widely observed in breast cancer and other cancer types. ZEB1 may interfere with these mechanisms by suppressing the expression of the miR‐200 family. Humulene, oleuropein, and rutin, alternatively, may increase ZEB1 expression, reducing the levels of miR‐200 family members, which could exhibit potential anti‐tumor effects against breast cancer. This process could be considered a significant strategy for the development of new therapeutic approaches.

Molecular docking studies of Humulene with 4P7U and 3NAR revealed strong binding interactions, with binding affinities of −7.5 and −7.1, respectively, indicating that Humulene is an effective molecule. Oleuropein, in its docking studies with 1D5R (−8.0), 7SJ3 (−9.2), and 3NAR (−6.1), also demonstrated strong binding, confirming its role as an efficient ligand. Rutin's docking studies with 1D5R (−8.4), 4P7U (−7.2), 7SJ3 (−9.2), 8T35 (−7.0), and 3NAR (−7.3) showed successful binding to these targets, highlighting its promising potential. Finally, Catechin's docking with 1D5R (−8.0), 7SJ3 (−9.3), and 8T35 (−7.4) demonstrated its strong binding capacity and confirmed its effectiveness as a ligand. Therefore, humulene, oleuropein, rutin, and catechin play a significant role in regulating miRNA expression through the pathways they affect.

## 4 Conclusions

GC–MS and HPLC‐DAD analyses revealed a rich phytochemical profile exhibiting significant biological activity, including important components such as 1S‐α‐pinene, humulene, caryophyllene, oleuropein, and catechin. The extract demonstrates potential as an anticancer agent by dose‐dependently inhibiting the viability of MDA‐MB‐231 cells. miRNA expression analysis showed a significant upregulation of miR‐19, miR‐20a, miR‐126, and miR‐200c in cancer cells, suggesting the extract's potential to regulate miRNA pathways in cancer treatment. In contrast, the extract caused downregulation of miRNAs in healthy control cells, highlighting its selective and distinct effects on cancerous and non‐cancerous cells. Molecular docking analyses comprehensively elucidated the anticancer effects, mRNA expression, and interactions between phytochemical compounds. Specifically, the anticancer activities of compounds such as rutin, oleuropein, catechin, and humulene were validated by their molecular binding potentials. miRNA expression reflects the biological activity of these compounds and their effects on target proteins, while molecular docking studies provide detailed insights into how these compounds bind to potential biological targets. These findings support the potential use of phytochemical compounds in anticancer therapies.

## Author Contributions

Z.B.S. and M.E.S.: conceptualization, data curation, formal analysis, writing – review. E.I.T.: formal analysis, investigation, methodology, resources, visualization, writing – original draft. E.C.A.: formal analysis, investigation, supervision, methodology, resources, software, writing – original draft, writing – review and editing. A.D. and A.G.: investigation, resources, validation. G.D.: investigation.

## Conflicts of Interest

The authors declare no conflicts of interest.

## Data Availability

Data will be made available on request.
